# The noncanonical RNA-binding protein RAN stabilizes the mRNA of intranuclear stress granule assembly factor G3BP1 in nasopharyngeal carcinoma

**DOI:** 10.1016/j.jbc.2024.107964

**Published:** 2024-11-05

**Authors:** Pan-Yang Yang, Zhenyu Yang, Jiawei Lv, Pei-Yi Jiang, Ting-Qiu Quan, Zhuo-Hui Huang, Xu-Dong Xu, Rui Guo, Denghui Wei, Ying Sun

**Affiliations:** 1 State Key Laboratory of Oncology in South China, Guangdong Key Laboratory of Nasopharyngeal Carcinoma Diagnosis and Therapy, Guangdong Provincial Clinical Research Center for Cancer, Sun Yat-sen University Cancer Center, Guangzhou, PR China; 2 State Key Laboratory of Oncology in South China, Guangdong Key Laboratory of Nasopharyngeal Carcinoma Diagnosis and Therapy, Guangdong Provincial Clinical Research Center for Cancer, Department of Radiation Oncology, Sun Yat-sen University Cancer Center, Guangzhou, PR China

## Abstract

RNA-binding proteins (RBPs) play critical roles in tumor progression by participating in the posttranscriptional regulation of RNA. However, the levels and function of RBPs in nasopharyngeal carcinoma (NPC) remain elusive. Here we identified a noncanonical RBP RAN that has the most significant role in NPC progression by a small siRNA pool screening. Functionally, RAN facilitates NPC proliferation and metastasis *in vitro* and *in vivo*. High levels of RAN are associated with poor prognosis of NPC patients and can be performed as a prognostic biomarker. Mechanistically, RAN increases the nucleus import of TDP43 and enhances TDP43 nuclear distribution. On the other hand, RAN is directly bound to the coding sequence of *G3BP1* mRNA and serves as an adapter to facilitate TDP43 interacting with *G3BP1* mRNA 3′ UTR. These contribute to increasing *G3BP1* mRNA stability in the nucleus and lead to upregulation of G3BP1, which further enhances AKT and ERK signaling and ultimately promotes NPC proliferation and metastasis. These findings reveal that RAN stabilizes intranuclear *G3BP1* mRNA by dual mechanisms: recruiting TDP43 into the nucleus and enhancing its interaction with *G3BP1* mRNA, suggesting a critical role of RAN in NPC progression and providing a new regulation framework of RBP-RNA.

RNA binding proteins (RBPs) are a type of proteins that can bind RNA and regulate RNA metabolism (1). The number of RBPs was extended to 1542 in 2014, about 75% of them are noncanonical RBPs which do not contain classical RNA-binding domains (2). RBPs are important participants in the posttranscriptional regulation of RNA and are closely related to the homeostasis of the intracellular environment, substance metabolism, and organization development (3). RBPs are widely involved in the stabilizing, splicing, translation, editing, and localization of RNA, which are indispensable factors for maintaining the normal life cycle of RNA (4). Thus, abnormal expression of RBPs can lead to extensive aberrations in RNA regulation and is closely associated with a variety of diseases (5). IGF2BP2 can bind and stabilize *TAB3* mRNA in an m6A-dependent manner, promoting acute kidney injury (6). RBP TDP43 directly interacts with N1-methyladenosine in RNA, which leads to its cytoplasmic mislocalization and induces neurodegenerative diseases (7).

Increasing evidence suggests that RBPs are aberrantly expressed in different types of tumors, RBPs play crucial roles in tumor progression and can also be used as a prognostic biomarker for tumors (8). Disturbance of the RBPs-RNA regulatory network is an important mechanism leading to tumor development (9). For example, estrogen receptor alpha can act as an RBP to regulate the posttranscriptional expression of stress response genes. Estrogen receptor alpha controls *XBP1* mRNA alternative splicing and promotes the translation of eIF4G2 and MCL1 mRNAs, ultimately facilitating breast tumor growth and enhancing therapeutic response (10). RBP RPS7 binds to the 3′ UTR of *LOXL2* mRNA and stabilizes it, further promoting hepatocellular carcinoma metastasis, which can perform as a prognostic biomarker for hepatocellular carcinoma patients (11).

Nasopharyngeal carcinoma (NPC) is a malignant tumor with Chinese endemic, and 47% of the global cases occur in China, among which the Guangdong region has the highest incidence (12). NPC is prevalent in young and middle-aged adults and can greatly impact family labor and socio-economics (13). Currently, distant metastasis is the main reason for treatment failure in NPC, about 70 percent (14). Once distant metastases are present, the median survival time for patients is only 20 months, even when treated with standard regimens (15). Studies have shown that RBPs are important in NPC progression and metastasis. MEX3A is proven to increase NPC cell proliferation and migration by interacting with hsa-miRNA-3163 and enhancing the level of SCIN (16). Through interacting with *JAK2* mRNA and then activating the JAK2/STAT3 signaling pathway, G3BP1 facilitates NPC progression and can be used as a biomarker for NPC patients' outcomes (17). IGF2BP3 can directly bind to lncRNA *TINCR* and inhibit its degradation, then elevate *TINCR* level and promote NPC development (18). However, the studies of RBPs in NPC are limited and lack systematic analysis, and none of the studies clarified the expression level of 1547 RBPs in NPC, and further detected the biological function of aberrantly expressed RBPs in NPC cell lines.

In the present study, we identified a noncanonical RBP RAN that was upregulated in NPC and had the most significant influence on NPC progression. RAN has a classical role in regulating the protein exchange between cytoplasm and nucleus that is associated with the development of several tumors (19). However, the posttranscriptional regulation mechanism of RAN and its role in NPC progression are still lacking. We discovered that RAN is associated with poor prognosis of NPC and facilitates NPC proliferation, migration, and invasion *in vitro* and *in vivo*. RAN increases the nucleus import of TDP43 and acts as an adapter to enhance its interaction with *G3BP1* mRNA, thus increasing the stability of intranuclear *G3BP1* mRNA and further enhancing AKT and ERK signaling, ultimately promoting NPC proliferation and metastasis.

## Results

### RAN is upregulated and associated with poor prognosis in NPC

**Figure 1:**
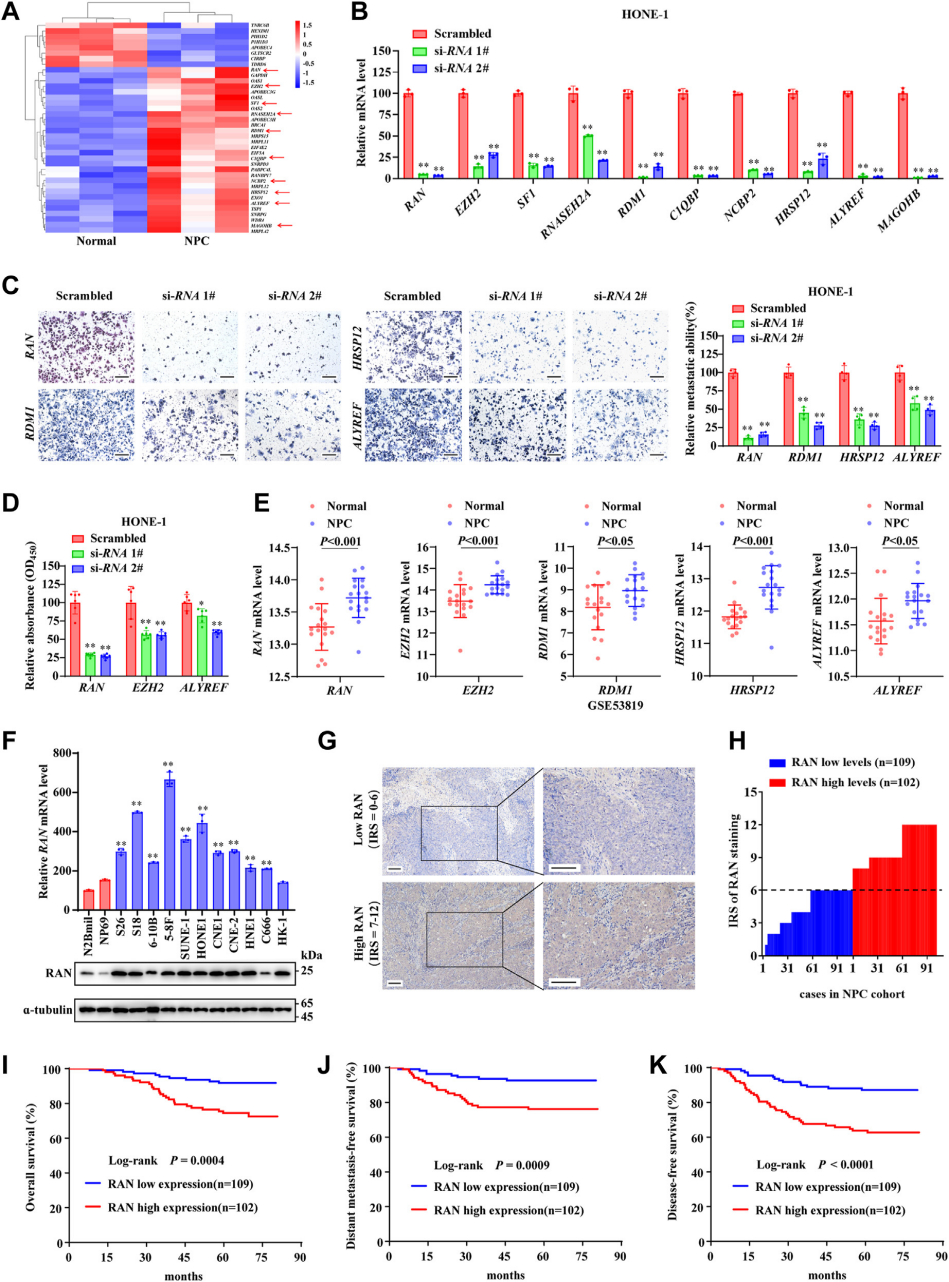
**Aberrant levels of RBPs are related to NPC progression.***A,* heat map was performed to illustrate the differently expressed RBPs between 3 NPC tissues and three normal nasopharyngeal tissues from our previous database (GSE126683). *B,* siRNA-mediated interference was used to knockdown selected RBPs in HONE-1 cells, and RT-qPCR was applied to determine knockdown efficiency. Data are presented as the mean ± SD (n = 3). *C,* representative images (*left*) and quantified results (*right*) of the transwell migration assays in HONE-1 cells. The scale bar represents 200 μm. Data are presented as the mean ± SD (n = 4). *D,* identification of cell proliferation ability by CCK-8 assays after 96 h of siRNA interference. Data are presented as the mean ± SD (n = 6). *E,* validation of *RAN*, *EZH2*, *RDM1*, *HRSP12*, and *ALYREF* expression levels in NPC tissues (n = 18) and normal nasopharyngeal tissues (n = 18) based on data from GEO database GSE53819. Data are presented as the mean ± SD (n = 18). *F,* evaluation of RAN expression level in two immortalized nasopharynx epidermal cells (N2Bmil and NP69) and NPC cell lines. RNA levels were indicated by RT-qPCR (*upper*), and protein levels were detected by Western blotting (*lower*). Data are presented as the mean ± SD (n = 3). *G*–*K,* the prognostic value of RAN expression levels in NPC was assessed by IHC staining in the NPC cohort (n = 211). *G,* RAN expression level was identified using the immunoreactivity score (IRS) system. Representative images of IHC staining for RAN were provided, and 0 to 6 were categorized as RAN low levels (*upper*), and 7 to 12 were categorized as RAN high levels (*lower*). The scale bar represents 100 μm. *H,* distribution of RAN IRS in the NPC cohort, consisted of RAN low levels (n = 109) and RAN high levels (n = 102). *I*–*K,* Kaplan–Meier curves of overall survival (*I*), distant metastasis-free survival (*J*), and disease-free survival (*K*) according to RAN levels. The log-rank test was used to compare differences in survival outcomes. ∗*p* < 0.05 and ∗∗*p* < 0.01. The significant differences were assessed using one-way ANOVA (*B–D* and *F*) and *t* test (*E*). CCK-8, Cell Counting Kit-8; RBP, RNA-binding protein; GEO, Gene Expression Omnibus; RT-qPCR, reverse transcription quantitative real-time PCR; IHC, immunohistochemistry; NPC, nasopharyngeal carcinoma.

To clarify the relationship between RBP levels and NPC progression, we analyzed the levels of the 1542 RBPs between three NPC tissues and three normal nasopharyngeal tissues from our previous database (GSE126683). The result showed that 30 RBPs were upregulated, and eight RBPs were downregulated in NPC (Fig. 1*A*). Next, the top 10 upregulated RBPs were individually knocked down using two siRNAs in HONE-1 cells (Fig. 1*B*). Transwell assays showed that silencing of RAN, RDM1, HRSP12, and ALYREF inhibited the migration of HONE-1 cells, while others did not (Fig. 1*C* and Fig. S1, *A* and *B*). Cell Counting Kit-8 (CCK-8) assays showed that silencing of RAN, EZH2, and ALYREF inhibited the proliferation of HONE-1 cells, while others did not (Fig. 1*D* and Fig. S1*C*). The aberrant upregulation of these 10 RBPs in NPC tissues was validated by another Gene Expression Omnibus (GEO) database consisting of 18 NPC tissues and 18 normal nasopharyngeal tissues (GSE53819). Nine of these RBPs, except SF1, were indeed elevated in NPC (Fig. 1*E* and Fig. S1*D*).

Since the upregulation of RAN in NPC tissues was confirmed by two individual GEO databases (GSE126683 and GSE53819), and knockdown of RAN had the most significant inhibition on both migration and proliferation of NPC cells, RAN might act as the most critical RBP in NPC progression, which needs further investigation. RAN has been reported to play an important role in the progression of several tumors (20, 21, 22), but its role in NPC remains elusive. RAN has also been identified to be a noncanonical RBP in several large-scale RBP census experiments (2, 23, 24), but the posttranscriptional regulation mechanism of RAN is still lacking.

To further investigate the expression level and clinical significance of RAN, we first showed that RAN expression levels were significantly elevated in NPC cell lines compared with two immortalized nasopharynx epidermal cells (N2Bmil and NP69), both in RNA and protein levels (Fig. 1*F*). We then analyzed the clinical significance of RAN in an NPC cohort (n = 211). The expression levels of RAN were detected by immunohistochemistry (IHC) staining and quantified by the immunoreactivity score (IRS) system (Fig. S2*A*). According to the IRS system, patients were divided into two groups: low levels of RAN (IRS = 0–6, n = 109) and high levels of RAN (IRS = 7–12, n = 102), (Fig. 1, *G* and *H*). High levels of RAN were significantly correlated with a poor clinical prognosis of NPC patients (Table S6). Kaplan–Meier analysis also showed that patients with higher RAN were associated with shorter overall survival (the period after treatment with death from any cause, Fig. 1*I*), distant metastasis-free survival (the period after treatment with no signs of cancer distant metastasis, Fig. 1*J*), and disease-free survival (the period after treatment with no signs of cancer recurrence, Fig. 1*K*). Multivariable Cox regression analysis showed that RAN levels, age, and TNM stage were independent prognostic factors for NPC patients (Fig. S2*B*). To further evaluate the prognostic value of RAN levels in NPC patients, we constructed an integrated risk prognostic model by combining RAN levels and TNM stage. NPC patients were divided into three groups, low risk (low levels of RAN and Ⅰ–III TNM stage, n = 75), medium risk (high levels of RAN or IV TNM stage, n = 90), and high risk (high levels of RAN and IV TNM stage, n = 46). Kaplan–Meier analysis revealed that the high-risk group was significantly correlated with death, distant metastasis, and tumor relapse (Fig. S2, *C*–*E*). These results suggested that RAN is upregulated in NPC and can be performed as a prognostic biomarker for NPC patients.

### RAN facilitates NPC proliferation and metastasis

**Figure 2:**
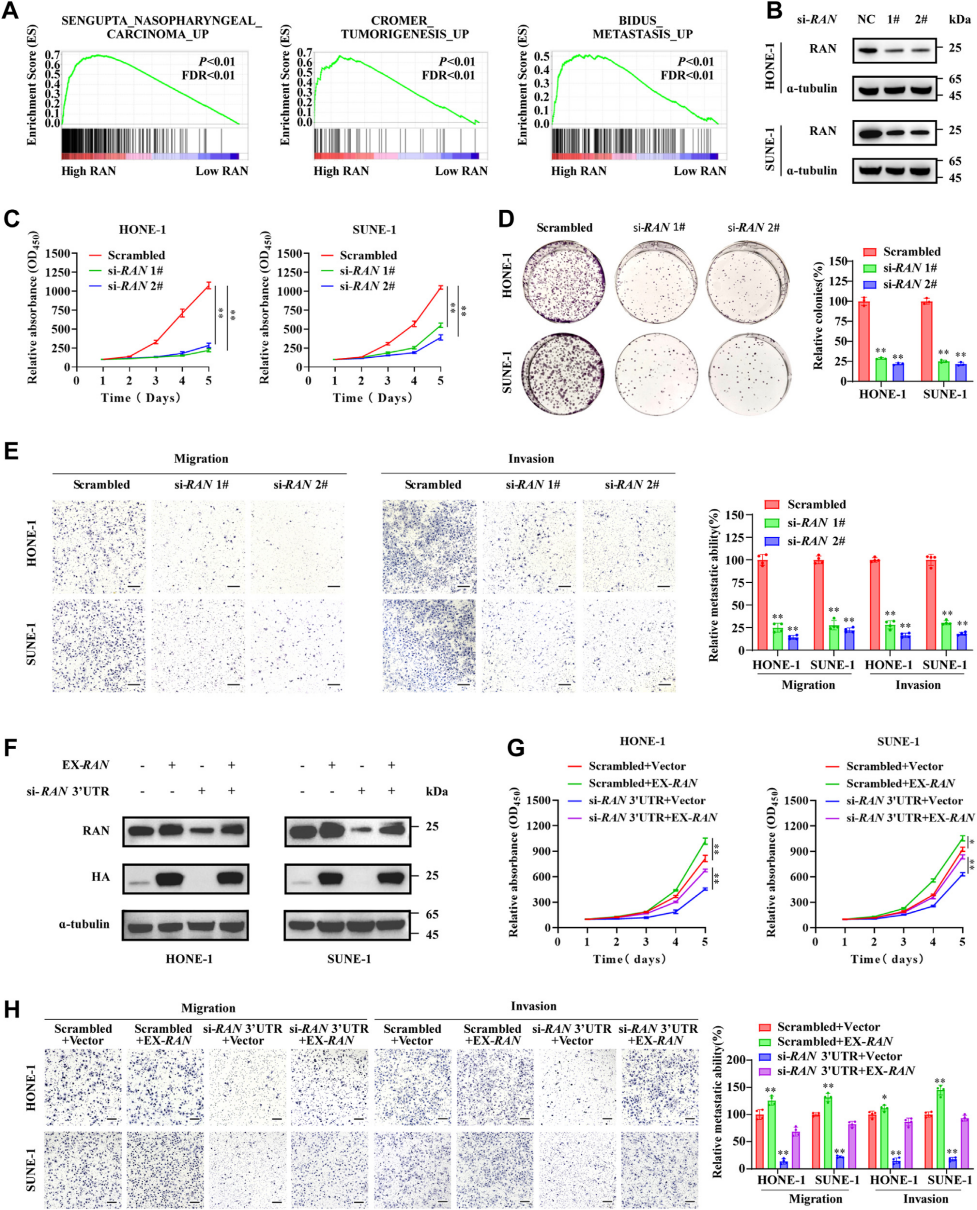
**RAN facilitates NPC cell proliferation, migration, and invasion *in vitro*.***A,* GSEA based on data from the GEO database (GSE53819) found that *RAN* expression levels were positively correlated with NPC progression and metastasis. *B,* siRNA-mediated interference was used to knockdown RAN in HONE-1 and SUNE-1 cells, and Western blotting was applied to validate knockdown efficiency. *C,* cell proliferation ability was evaluated by CCK-8 assays in HONE-1 and SUNE-1 cells after silencing of RAN. Data are presented as the mean ± SD (n = 6). *D,* cell proliferation ability was evaluated by colony formation assays in HONE-1 and SUNE-1 cells after silencing of RAN. Data are presented as the mean ± SD (n = 3). *E,* migration and invasion capacity were analyzed by transwell assays in RAN-silenced SUNE-1 and HONE-1 cells. The scale bar represents 200 μm. Data are presented as the mean ± SD (n = 4). *F,* Western blotting was applied to confirm the cotransfection efficiency of scrambled control or siRNA-targeting RAN 3′ UTR, together with empty vector or HA-tagged RAN overexpression vector. *G,* cell proliferation was evaluated by CCK-8 assays in SUNE-1 and HONE-1 cells after cotransfected with scrambled control or si-RAN 3′ UTR, together with empty vector or HA-tagged RAN overexpression vector. Data are presented as the mean ± SD (n = 6). *H,* migration and invasion capacity were analyzed by transwell assays in SUNE-1 and HONE-1 cells after cotransfected with scrambled control or si-RAN 3′ UTR, together with empty vector or HA-tagged RAN overexpression vector. Data are presented as the mean ± SD (n = 4). ∗*p* < 0.05, ∗∗*p* < 0.01. The significant differences were assessed using one-way ANOVA (*D*, *E*, and *H*) and two-way ANOVA (*C* and *G*). CCK-8, Cell Counting Kit-8; GEO, Gene Expression Omnibus; NPC, nasopharyngeal carcinoma; GSEA, gene set enrichment analysis; HA, hemagglutinin.

To further investigate the biological function of RAN in NPC, we first performed a gene set enrichment analysis based on data from the GEO database GSE53819. The result showed that expression levels of *RAN* were positively correlated with NPC progression and metastasis (Fig. 2*A*). We then silenced RAN in HONE-1 and SUNE-1 cells using two individual siRNAs (si-*RAN* 1# and si-*RAN* 2#, Fig. 2*B*). CCK-8 assays and colony formation assays showed that knockdown of RAN impaired the proliferation ability of HONE-1 and SUNE-1 cells (Fig. 2, *C* and *D*). Transwell assays showed that silencing of RAN inhibited the migration and invasion capacity of NPC cells (Fig. 2*E*). To assess off-target effects of siRNAs, we overexpressed RAN tagged with Human influenza hemagglutinin in cells and silenced endogenous RAN by siRNA-targeting 3′ UTR of RAN mRNA. The exogenous RAN overexpression could not be impaired by si-RAN 3′ UTR (Fig. 2*F*). Subsequently, CCK-8 and transwell assays showed that overexpression of exogenous RAN rescued the impaired proliferation, migration, and invasion ability of NPC cells with endogenous RAN silencing (Fig. 2, *G* and *H*). Altogether, these findings suggested that RAN facilitates NPC proliferation, migration, and invasion *in vitro*.

**Figure 3:**
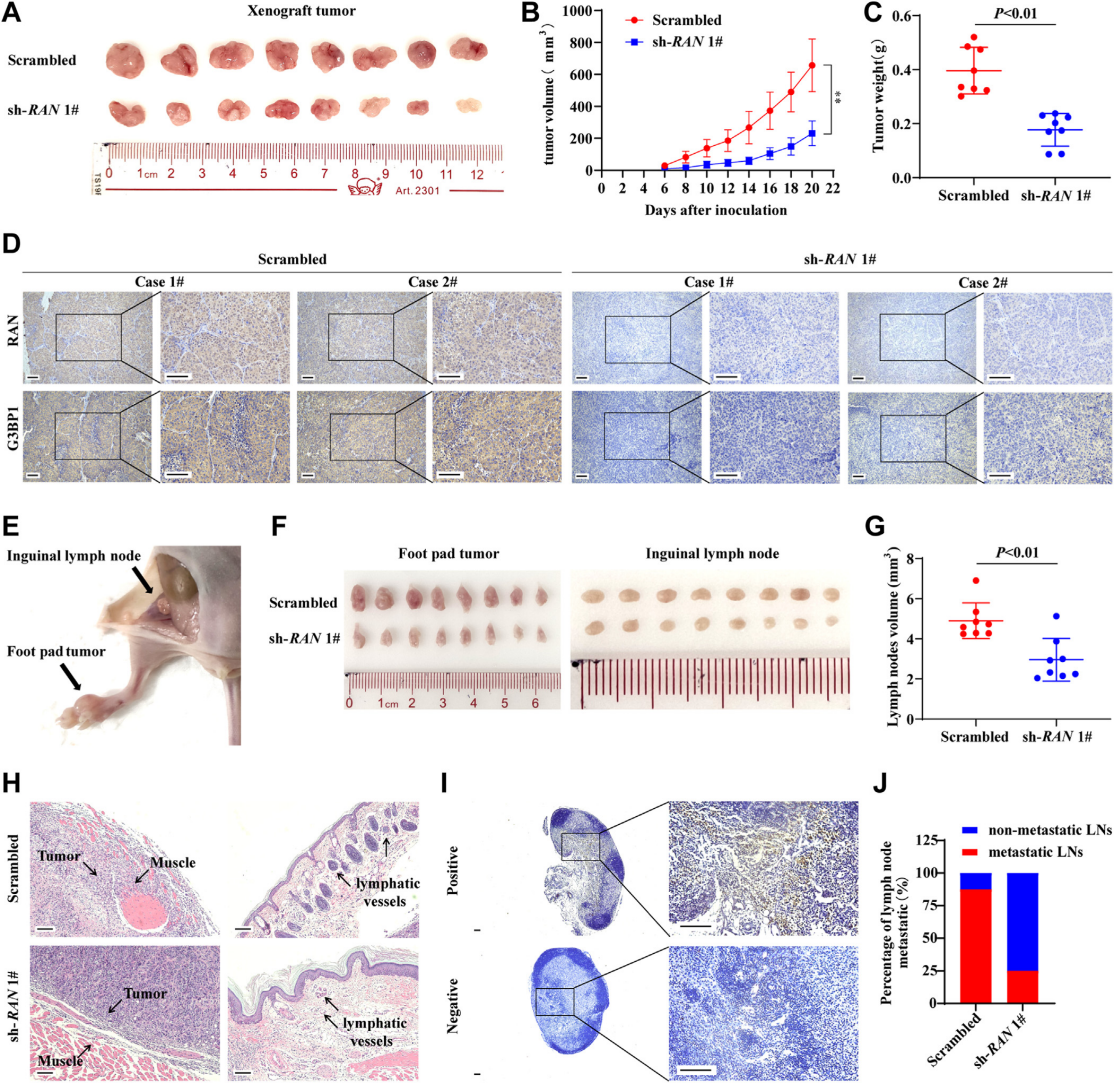
**Silencing RAN impairs NPC proliferation and invasion *in vivo***. *A–D,* SUNE-1 cells stably transfected with scrambled control shRNA or sh-*RAN* 1# were subcutaneously injected in nude mice to construct a xenograft tumor model. *A,* a representative image of the xenograft tumors. B, the tumor growth curves of the xenografts. Data are presented as the mean ± SD (n = 8). *C,* the tumor weights of the xenografts. Data are presented as the mean ± SD (n = 8). *D,* xenograft tumors were paraffin-embedded and sectioned. Then, the expression levels of RAN and G3BP1 were assessed by IHC staining. The scale bar represents 100 μm. *E–J,* SUNE-1 cells with or without RAN stable silencing were injected into the footpad of nude mice to establish an inguinal lymph node metastasis model. *E,* representative image of the inguinal lymph node metastasis model. *F,* representative images of the primary footpad tumors (*left*) and metastatic inguinal lymph nodes (*right*). *G,* the volume of the inguinal lymph nodes was calculated. Data are presented as the mean ± SD (n = 8). *H,* representative images of footpad tumor sections stained with H&E showing tumor cells invasion into muscle tissues (*left*) or lymphatic vessels (*right*). The scale bar represents 100 μm. *I,* representative images of IHC staining with pan-cytokeratin in metastatic (*upper*) or nonmetastatic (*lower*) inguinal lymph nodes. The scale bar represents 100 μm. *J,* the quantitative result of the ratios of inguinal lymph nodes metastasis. ∗*p* < 0.05 and ∗∗*p* < 0.01. The significant differences were assessed using two-way ANOVA (*A*) and *t* test (*B* and *G*). GEO, Gene Expression Omnibus; RT-qPCR, reverse transcription quantitative real-time PCR; IHC, immunohistochemistry; NPC, nasopharyngeal carcinoma.

The functions of RAN are further investigated *in vivo* by establishing a xenograft tumor model and an inguinal lymph node metastasis model. SUNE-1 cells stably transfected with scrambled control shRNA or sh-RAN were constructed. Xenograft tumors of nude mice subcutaneously injected with RAN-silenced cells grew slower and had lower tumor volume and weight (Fig. 3, *A*–*C*). Knockdown efficiency of RAN was verified by IHC (Fig. 3*D*). Furthermore, nude mice whose footpads were injected with RAN-silenced cells have smaller volumes of footpad tumors and inguinal lymph nodes(Fig. 3, *E*–*G*). H&E staining of footpad tumor sections showed that the tumor is well demarcated from the muscle tissue and lymphatic vessels maintained simple epithelium after RAN silencing, suggesting that knockdown of RAN inhibited tumor invasion from footpad into muscle tissues and lymphatic vessels (Fig. 3*H*). Inguinal lymph nodes were stained by pan-cytokeratin to detect metastatic tumor cells. Less metastatic inguinal lymph nodes were detected in the RAN-silenced group (Fig. 3, *I* and *J*). Collectively, RAN facilitates the proliferation and metastasis of NPC *in vitro* and *in vivo*.

### RAN binds and stabilizes *G3BP1* mRNA

**Figure 4:**
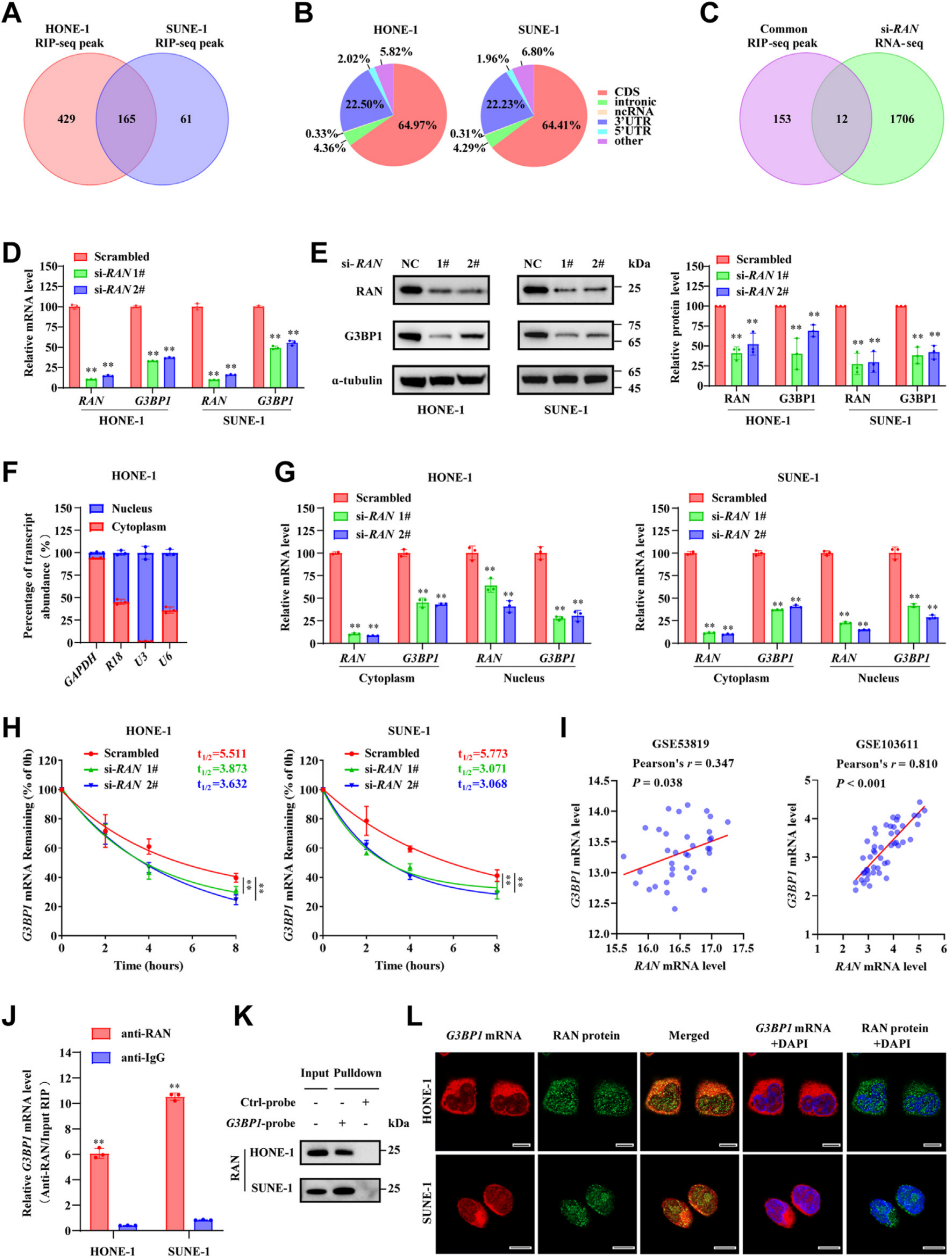
**RAN directly binds and stabilizes *G3BP1* mRNA.***A,* Venn diagram of genes identified by RIP-seq in HONE-1 or SUNE-1 NPC cell lines (logOddScore >1). *B,* pie charts showing the distribution of reads recognized by RIP-seq on gene functional elements. *C,* venn diagram of differentially expressed genes identified by RNA-seq after RAN silencing and overlapping genes identified by RIP-seq. *D,* relative levels of *G3BP1* mRNA level with or without RAN silencing were detected by RT-qPCR. Data are presented as the mean ± SD (n = 3). *E,* G3BP1 protein level with or without RAN silencing was detected by Western blotting. The blots are the representation of three independent experiments. Data are presented as the mean ± SD (n = 3). *F,* nuclear/cytosol RNA fractionation assays were used to identify the suitable internal control. Data are presented as the mean ± SD (n = 3). *G,* relative levels of *G3BP1* mRNA level in cytoplasm or nucleus, which were normalized to *GAPDH* or *U3*, was indicated by RT-qPCR upon knockdown of RAN in HONE-1 and SUNE-1 cells. Data are presented as the mean ± SD (n = 3). *H,* after treatment with actinomycin D (10 μg/ml), *G3BP1* mRNA level was quantified at indicated times in control and RAN-silenced cells. The half-life of *G3BP1* mRNA was analyzed by plotting degradation curves. Data are presented as the mean ± 95%CI (n = 3). *I,* Pearson correlation analysis of *RAN* and *G3BP1* levels in different GEO databases (GSE53819 and GSE103611). *J,* relative enrichment of *G3BP1* mRNA immunoprecipitated by anti-RAN or anti-IgG antibody was indicated by RT-qPCR. Data are presented as the mean ± SD (n = 3). *K,* enrichment of RAN proteins pulled down by biotin-labeled *G3BP1* probes from *in vitro* transcription or control antisense probes was detected by Western blotting. *L,* RAN protein distribution in cells was recognized by IF (*green*), *G3BP1* mRNA distribution in cells was recognized by FISH (*red*), and cell nuclei were stained with DAPI (*blue*). The scale bar represents 10 μm. ∗*p* < 0.05 and ∗∗*p* < 0.01. The significant differences were assessed using one-way ANOVA (*D*, *E*, and *G*), two-way ANOVA (*H*), and *t* test (*J*). The significant differences in correlations were assessed using the Pearson correlation analysis (*I*). GEO, Gene Expression Omnibus; RT-qPCR, reverse transcription quantitative real-time PCR; NPC, nasopharyngeal carcinoma; DAPI, 4′, 6-diamino-2-phenylindole; IF, immunofluorescence; CI, confidence interval.

Since RAN is a noncanonical RBP facilitating the proliferation and metastasis of NPC, we urgently need to identify the RNAs specifically bound by RAN. To obtain more accurate data, RNA immunoprecipitation sequencing (RIP-seq) was conducted in two NPC cell lines HONE-1 and SUNE-1, respectively. RIPSeeker software package was used to identify protein-associated transcripts (http://www.bioconductor.org/packages/release/bioc/html/RIPSeeker.html) (25). Transcripts of 165 genes could specifically bind to RAN in both HONE-1 and SUNE-1 cells (logOddScore > 1, Fig. 4*A*). Kyoto Encyclopedia of Genes and Genomes pathway analysis showed that the 165 overlapping genes were enriched in cancer-related pathways, suggesting that RAN may promote NPC development by its RNA binding ability (Fig. S3*A*). Since the regulation of nucleo-cytoplasmic substance transport is an important function of RAN (19), we first detected whether RAN silencing could lead to nuclear retention of transcripts of the top eight genes identified by RIP-seq. Disappointingly, we did not detect elevated RNA levels in the nucleus, as well as decreased RNA levels in the cytoplasm after RAN silencing (Fig. S3, *B*–*D*). These results suggested that RAN may be unable to regulate mRNA nuclear export, consistent with recent research (26).

Due to the preference for RAN binding to the coding sequence (CDS) and the 3′ UTR of transcripts (Fig. 4*B*), RNA-seq was performed to search transcripts bound and regulated by RAN. After knockdown of RAN, 1718 differentially expressed genes were detected (|log2(fold change (FC))| >1 and *p* < 0.05, Fig. S4*A*). The Gene Oncology analyses, Kyoto Encyclopedia of Genes and Genomes pathway analysis, and gene set enrichment analysis of data from RNA-seq further confirmed the important role of RAN in NPC development (Fig. S4, *C*–*G*). Twelve of 1718 genes could be found in the RIP-seq data of HONE-1 and SUNE-1 cells (Fig. 4*C* and Fig. S4*B*), among which eight genes were downregulated and four genes were upregulated. The RNA expression levels of eight downregulated genes were verified by reverse transcription quantitative real-time PCR (RT-qPCR) in RAN-silenced HONE-1 and SUNE-1 cells, and the expression level of *G3BP1* was most significantly decreased (Fig. S5*A*).

G3BP1 has multiple important roles in tumor progression (27). Knockdown of RAN significantly inhibited the RNA and protein expression levels of G3BP1 (Fig. 4, *D* and *E*). Since RAN is distributed both inside and outside the nucleus, we isolated the nucleus and cytoplasm RNA to identify the location of *G3BP1* mRNA regulated by RAN, *GAPDH* was selected as cytoplasm internal control and *U3* was selected as nucleus internal control (Fig. 4*F*). Silence of RAN decreased the levels of *G3BP1* mRNA both in the cytoplasm and the nucleus (Fig. 4*G*). Moreover, *G3BP1* mRNA degraded more quickly and had a shorter half-life in RAN-silenced HONE-1 and SUNE-1 cells after treatment with actinomycin D (Fig. 4*H*). G3BP1 levels assessed by IHC staining were also remarkably decreased in xenograft tumors of the RAN-silenced group (Fig. 3*D*). Correspondingly, the levels of *RAN* were positively correlated with *G3BP1* levels in several GEO databases based on RNA-seq from NPC tissues (Fig. 4*I* and Fig. S5*B*). These findings suggested that RAN increases *G3BP1* mRNA stability and leads to upregulation of G3BP1.

RIP-qPCR assay by anti-RAN or anti-IgG antibodies confirmed that RAN can specifically bind *G3BP1* mRNA (Fig. 4*J*). The interaction of RAN protein and *G3BP1* mRNA was further verified by using biotin-labeled *G3BP1* mRNA probes from *in vitro* transcription or control antisense probes. RAN was specifically pulled down by *G3BP1* mRNA probes rather than antisense probes detected by silver staining and Western blotting (Fig. 4*K* and Fig. S6*A*). The intracellular distribution of RAN proteins was recognized by immunofluorescence (IF) and the distribution of *G3BP1* mRNA was recognized by FISH. The colocalization of RAN and *G3BP1* mRNA was observed by confocal microscopy in the cytoplasm and the nucleus of HONE-1 and SUNE-1 cells (Fig. 4*L*). Radial line profile analysis also showed that the fluorescence intensity of RAN and *G3BP1* mRNA displayed a similar distribution pattern (Fig. S5*C*). Taken together, these results indicated that RAN can bind to *G3BP1* mRNA and maintain its stabilization, further leading to the elevation of the G3BP1 protein level.

### RAN, TDP43, and *G3BP1* mRNA form a complex in the nucleus

**Figure 5:**
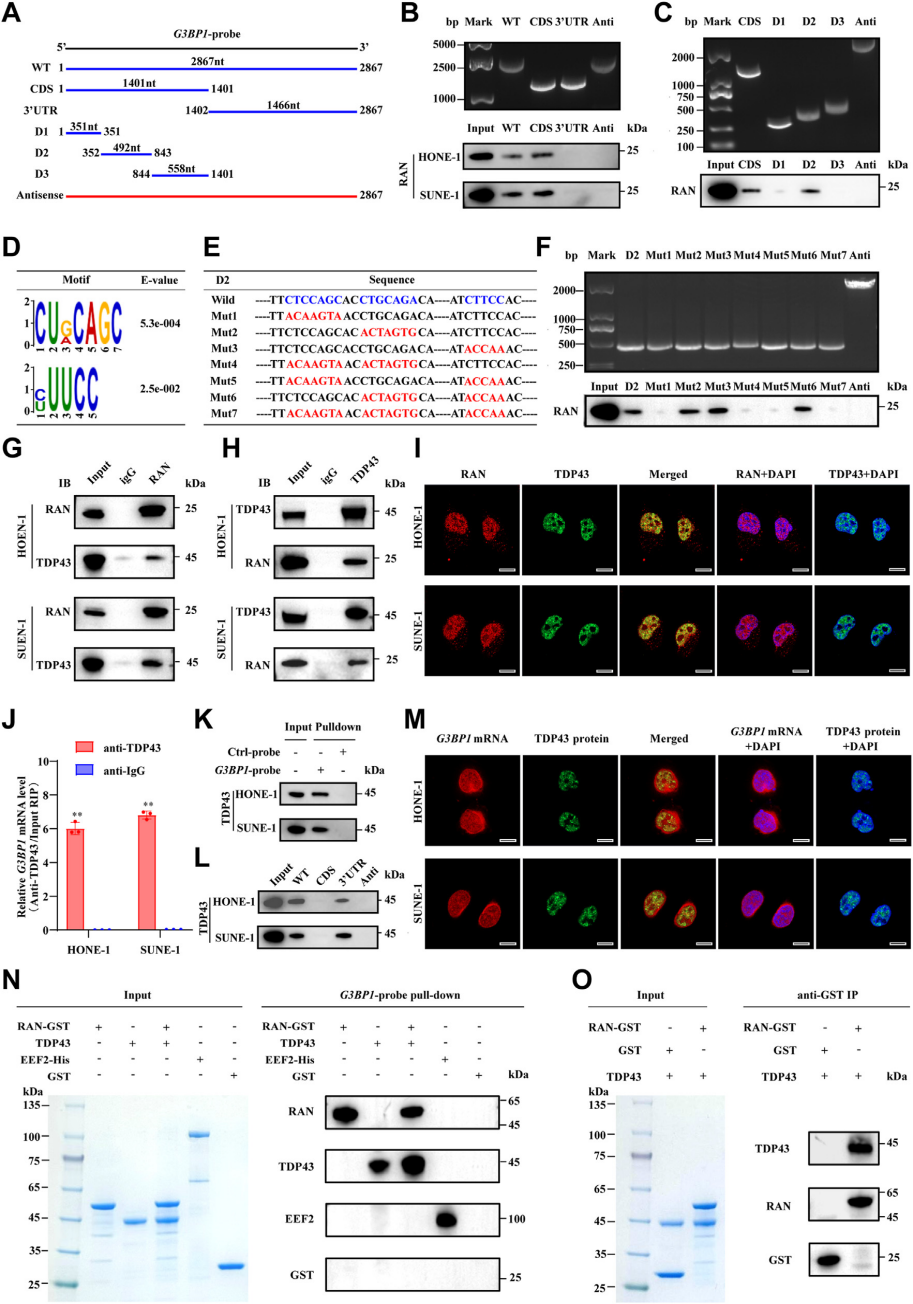
**RAN, TDP43, and *G3BP1* mRNA form a complex in the nucleus.***A,* diagrams of full-length *G3BP1* transcript consist of CDS and 3′ UTR, as well as its deletion fragments. *B* and *C,* the *in vitro*–transcribed full-length G3BP1 transcript and deletion fragments with the correct sizes were indicated (*upper*). RNA pull-down assay and Western blotting showed whether these biotin-labeled *G3BP1* fragments could pull down RAN in cell lysates (*lower*). *D,* the motif discovery algorithm DREME was used to recognize the top consensus motif based on the RIP-seq data. *E,* construction of mutant fragments based on predicted motifs. *F,* the *in vitro*–transcribed D2 fragments and mutant fragments with the correct sizes are indicated (*upper*). RNA pull-down assay and Western blotting indicated whether these biotin-labeled fragments could pull down RAN in cell lysates (*lower*). *G,* the interaction between endogenous RAN and TDP43 was evaluated by Co-IP assays using anti-RAN antibody or normal rabbit IgG in HONE-1 and SUNE-1 cell lysates. *H,* the interaction between endogenous TDP43 and RAN was evaluated by Co-IP assays using anti-TDP43 antibody or normal rabbit IgG in HONE-1 and SUNE-1 cell lysates. *I,* RAN (*red*) or TDP43 (*green*) protein distribution in cells was recognized by IF, and cell nuclei were stained with DAPI (*blue*). The scale bar represents 10 μm. *J,* relative enrichment of *G3BP1* mRNA immunoprecipitated by anti-TDP43 or anti-IgG antibody was indicated by RT-qPCR. Data are presented as the mean ± SD (n = 3). *K,* enrichment of TDP43 pulled down by biotin-labeled *G3BP1* probes from *in vitro* transcription or control antisense probes was detected by Western blotting. *L,* RNA pull-down assay and Western blotting showed whether biotin-labeled full-length G3BP1 transcript or deletion fragments could pull down TDP43 in cell lysates. *M,* TDP43 protein distribution in cells was recognized by IF (*green*), *G3BP1* mRNA distribution in cells was recognized by FISH (*red*), and cell nuclei were stained with DAPI (*blue*). The scale bar represents 10 μm. *N, in vitro* purified RAN-GST, TDP43, eEF2-His (positive control), and GST (negative control) proteins were used to perform a cell-free RNA pull-down experiment. Coomassie brilliant blue staining showed the purification of these proteins (*left*). Enrichment of proteins pulled down by biotin-labeled *G3BP1* probes from *in vitro* transcription was detected by Western blotting (*right*). *O, in vitro* purified RAN-GST, TDP43, and GST (negative control) proteins were used to perform a cell-free co-IP experiment. Coomassie brilliant blue staining showed the purification of these proteins (*left*). Enrichment of proteins immunoprecipitated by anti-GST magnetic beads was detected by Western blotting (*right*). ∗*p* < 0.05 and ∗∗*p* < 0.01. The significant differences were assessed using *t* test (*J*). RT-qPCR, reverse transcription quantitative real-time PCR; ; NPC, nasopharyngeal carcinoma; Co-IP, coimmunoprecipitation; GST, glutathione-*S*-transferase; IF, immunofluorescence; DAPI, 4′, 6-diamino-2-phenylindole; CDS, coding sequence.

To clarify the specific binding region between RAN and *G3BP1* mRNA, we constructed *G3BP1* mRNA deletion fragments of different lengths step by step (Fig. 5*A*). Surprisingly, RNA pull-down assays showed that RAN tended to bind the CDS of *G3BP1* mRNA rather than its 3′ UTR (Fig. 5*B*). These results suggested that RAN may not directly regulate *G3BP1* mRNA stability (28), but may act as an adapter to promote another factor binding to *G3BP1* mRNA. Based on the *G3BP1* gene exons, 3 shorter deletion fragments of the *G3BP1* mRNA CDS were constructed (Fig. S6*B*). The secondary structure and minimum free energy structure of the *G3BP1* mRNA CDS were predicted by using the online tool RNAfold WebServer (http://rna.tbi.univie.ac.at/) (Fig. S6*C*). RNA pull-down and Western blotting assays showed that the 352- to 843-nt region of *G3BP1* mRNA was necessary for its interaction with RAN, which is consistent with website predictions (Fig. 5*C*). The motif discovery algorithm DREME (https://meme-suite.org/meme/tools/dreme) was used to recognize the top consensus motif based on the RIP-seq data (Fig. 5*D*). Then seven mutant fragments were constructed based on predicted motifs (Fig. 5*E*). Interestingly, among seven mutant fragments, only mutating CTCCAGC sequence to ACAAGTA (A to G, G to T, T to C, C to A) impaired the pull down of RAN (Mut1, Mut4, Mut5, and Mut7), suggested that the CUNCAGC motif (N corresponding to A, U, C, G) of *G3BP1* mRNA was necessary for its interaction with RAN (Fig. 5*F*).

To identify the candidate that directly stabilizes *G3BP1* mRNA, the enriched proteins pulled down by *G3BP1* probes and anti-RAN antibody were subjected to mass spectrometry (MS) analysis. Both analyses identified the TDP43 protein (Fig. S6, *D* and *E*), which was reported to regulate mRNA stability by directly binding to the 3′ UTR (29). Western blotting confirmed that TDP43 was immunoprecipitated by anti-RAN antibody in HONE-1 and SUNE-1 cells (Fig. 5*G*). Coimmunoprecipitation (Co-IP) assays also showed that RAN could be immunoprecipitated by anti-TDP43 antibody (Fig. 5*H*). A remarkable colocalization of RAN and TDP43 in the nucleus can be observed by IF in HONE-1 and SUNE-1 cells (Fig. 5*I*). Radial line profile analysis also showed that the fluorescence intensity of RAN and TDP43 displayed a similar distribution pattern (Fig. S6*F*). We then verified the interaction of TDP43 with *G3BP1* mRNA. RIP-qPCR assay by anti-TDP43 confirmed that TDP43 can specifically bind *G3BP1* mRNA (Fig. 5*J*). Proteins were enriched by RNA pull-down assays and detected by silver staining and Western blotting, TDP43 was specifically pulled down by *G3BP1* mRNA probes rather than antisense probes (Figs. 5*K* and Fig. S6*A*). As expected, RNA pull-down assays showed that TDP43 tended to bind the 3′ UTR of *G3BP1* mRNA rather than its CDS (Fig. 5*L*). FISH accompanied by IF showed that TDP43 colocalized with *G3BP1* mRNA in the nucleus of HONE-1 and SUNE-1 cells (Fig. 5*M*). Radial line profile analysis also showed that the fluorescence intensity of RAN and TDP43 displayed a similar distribution pattern (Fig. S6*G*).

To further verify whether *G3BP1* mRNA is directly bound to RAN and TDP43, *in vitro* purified RAN-glutathione-*S*-transferase (GST) and TDP43 proteins were used to perform a cell-free RNA pull-down experiment. *In vitro* purified eEF2-His protein, which has a broad mRNA binding capacity (30), acted as the positive control, and *in vitro* purified GST protein acted as the negative control. Western blotting showed that RAN, TDP43, and eEF2 proteins were pulled down by biotin-labeled *G3BP1* probes from *in vitro* transcription, but GST protein was not. Additionally, the addition of RAN enhanced the enrichment of TDP43 pulled down by *G3BP1* mRNA. These suggested that both RAN and TDP43 bind directly to *G3BP1* mRNA and RAN increases the binding of TDP43 to *G3BP1* mRNA (Fig. 5*N*). A cell-free co-IP assay using anti-GST magnetic beads also showed that *in vitro* purified TDP43 protein could be immunoprecipitated by *in vitro* purified RAN-GST protein but not GST. It suggested that RAN binds directly to TDP43 (Fig. 5*O*). These results demonstrated that RAN, TDP43, and *G3BP1* mRNA can directly bind with each other to form a complex in the nucleus, thereby maintaining the stability of intranuclear *G3BP1* mRNA.

### RAN increases the nucleus import of TDP43 and facilitates TDP43 binding to *G3BP1* mRNA

To verify the regulatory relationship between TDP43 and *G3BP1* mRNA, *G3BP1* mRNA expression levels in whole-cell lysate, cytoplasm, and nucleus were detected upon knockdown of TDP43 in HONE-1 and SUNE-1 cells. The result showed that knockdown of TDP43 decreased the levels of *G3BP1* mRNA in the nucleus, which led to decreased cytoplasmic and whole-cell *G3BP1* mRNA expression levels (Fig. S7*A*). *G3BP1* mRNA degraded more quickly and had a shorter half-life in TDP43-silenced HONE-1 and SUNE-1 cells after treatment with actinomycin D (Fig. S7*B*). Knockdown of TDP43 also significantly inhibited the protein levels of G3BP1 in HONE-1 and SUNE-1 cells (Fig. S7*C*). Consistent with these results, the levels of *TDP43* were positively correlated with *G3BP1* levels in several GEO databases (Fig. S7*D*). We simultaneously explored the biological function of TDP43 in NPC. The knockdown efficiency of TDP43 is verified by RT-qPCR and Western blotting (Fig. S8, *A* and *B*). CCK-8 assays and colony formation assays showed that knockdown of TDP43 impaired the proliferation ability of HONE-1 and SUNE-1 cells (Fig. S8, *C* and *D*). Transwell assays showed that TDP43 silencing inhibited the migration and invasion capacity of NPC cells (Fig. S8*E*).

**Figure 6:**
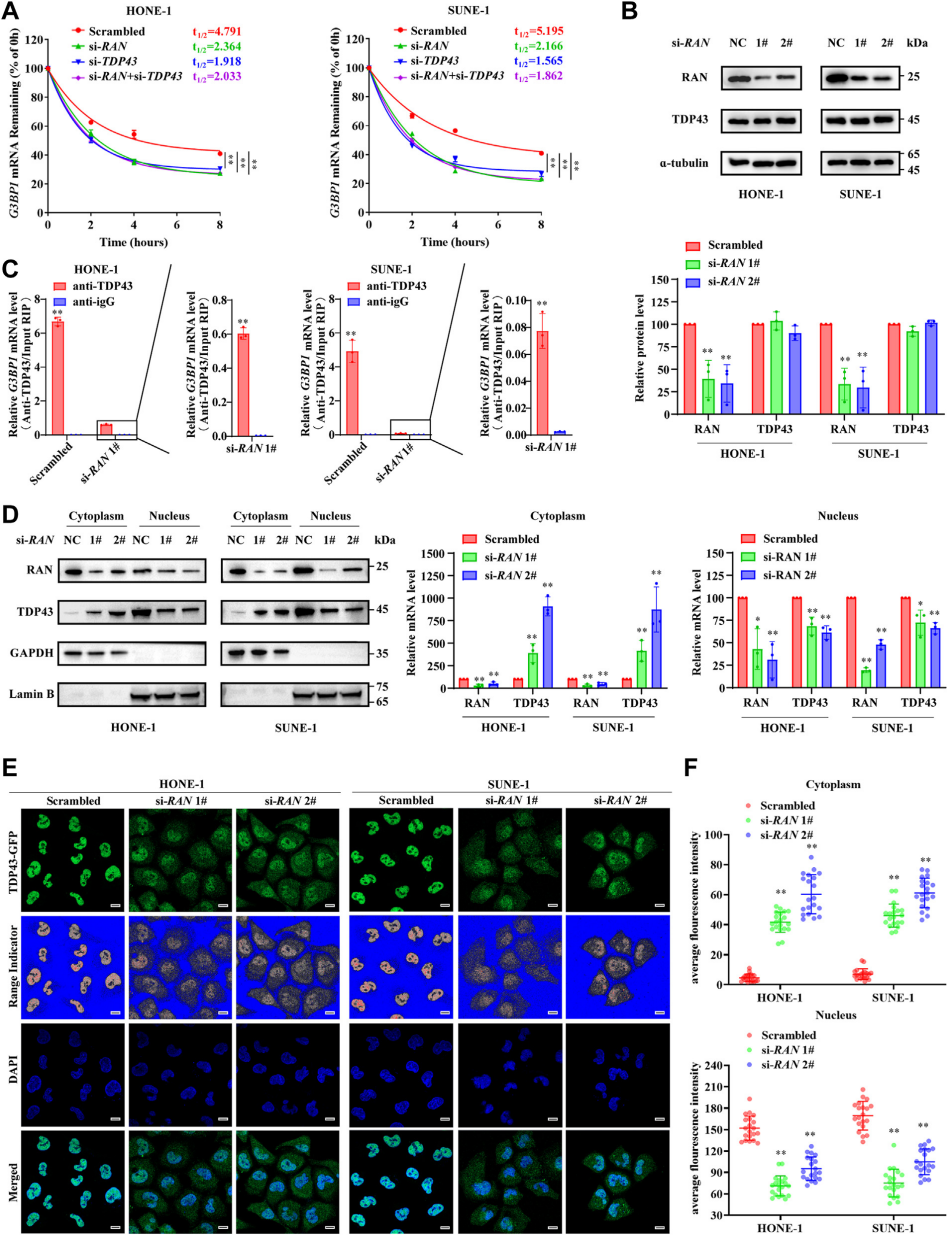
**RAN increases TDP43 nucleus import and facilitates TDP43 binding *G3BP1* mRNA.***A,* after treatment with actinomycin D (10 μg/ml), *G3BP1* mRNA level was quantified at indicated times in control, RAN-silenced, TDP43-silenced, and both RAN and TDP43-silenced cells. The half-life of *G3BP1* mRNA was analyzed by plotting degradation curves. Data are presented as the mean ± 95%CI (n = 3). *B,* TDP43 protein levels with knockdown of RAN were detected by Western blotting. The blots are the representation of three independent experiments. Data are presented as the mean ± SD (n = 3). *C,* relative enrichment of *G3BP1* mRNA immunoprecipitated by anti-TDP43 or anti-IgG antibody was indicated by RT-qPCR upon knockdown of RAN. Data are presented as the mean ± SD (n = 3). *D,* the nucleus and cytoplasm proteins in HONE-1 and SUNE-1 cells were isolated by nuclear/cytosol fractionation assays. TDP43 distribution in the nucleus or cytoplasm was identified by Western blotting with or without RAN silencing, GAPDH as the cytoplasm marker, and Lamin B as the nucleus marker. The blots are the representation of three independent experiments. Data are presented as the mean ± SD (n = 3). *E,* TDP43-GFP overexpression vectors were transfected in HONE-1 and SUNE-1 cells after RAN silencing. Recognizing TDP43-GFP protein distribution (*green*) in HONE-1 and SUNE-1 cells after 24 h of TDP43-GFP overexpression vectors transfected, range indicator was performed to show clearer distribution of TDP43-GFP in the nucleus or cytoplasm, cell nuclei were stained with DAPI (*blue*). The scale bar represents 10 μm. *F,* the average fluorescence intensity of TDP43-GFP in the nucleus (*lower*) and cytoplasm (*upper*) of each cell was calculated. Data are presented as the mean ± SD (n = 20). ∗*p* < 0.05 and ∗∗*p* < 0.01. The significant differences were assessed using one-way ANOVA (*B*, *D*, and *F*), two-way ANOVA (*A*), and *t* test (*C*). RT-qPCR, reverse transcription quantitative real-time PCR; DAPI, 4′, 6-diamino-2-phenylindole; CI, confidence interval.

To verify RAN stabilizes *G3BP1* mRNA *via* TDP43, we simultaneously silenced RAN and TDP43 in HONE-1 and SUNE-1 cells. Simultaneously silencing RAN and TDP43 did not further decrease the stability of G3BP1 compared to silencing TDP43 simply. It suggested that RAN is dependent on TDP43 to stabilize *G3BP1* mRNA (Fig. 6*A*). However, knockdown of RAN did not affect TDP43 levels (Fig. 6*B*). After RNA immunoprecipitating by anti-TDP43 antibody with or without RAN silencing, the relative enrichment of *G3BP1* mRNA was detected by RT-qPCR. RAN silencing resulted in a 10- to 50-fold reduction in *G3BP1* mRNA enrichment in HONE-1 and SUNE-1 cells, indicating a crucial role for RAN in the binding of TDP43 to *G3BP1* mRNA (Fig. 6*C*). RAN has an extensive role in nucleo-cytoplasmic transport of proteins (31), and TDP43 nuclear location is vital for its function (32), so we isolated the nucleus and cytoplasm proteins in HONE-1 and SUNE-1 cells by nuclear/cytosol fractionation assays. Knockdown of RAN increased TDP43 in the cytoplasm and decreased TDP43 in the nucleus, suggesting that knockdown of RAN leads to cytoplasmic retention of TDP43 (Fig. 6*D*). Subsequently, the intracellular distribution of TDP43 was detected by IF in HONE-1 and SUNE-1 cells. TDP43 was completely distributed in the nucleus of control cells, but more TDP43 could be observed in the cytoplasm of RAN-silenced cells (Fig. S7*E*). After calculating the average fluorescence intensity of TDP43 in the nucleus or cytoplasm per cell, we confirmed that knockdown of RAN increased the cytoplasmic distribution of TDP43 and decreased the TDP43 nucleus distribution (Fig. S7*F*). Then, to avoid the influence of TDP43 which had already entered the nucleus before RAN silencing, TDP43-GFP overexpression vectors were transfected in HONE-1 and SUNE-1 cells after RAN silencing for 24 h after TDP43-GFP transfection, the intracellular distribution of TDP43-GFP was observed by confocal microscopy. A significantly increasing distribution of TDP43-GFP in the cytoplasm and a significantly decreasing distribution of TDP43-GFP in the nucleus could be observed in RAN-silenced cells (Fig. 6*E*). This result was also confirmed by calculating the average fluorescence intensity of TDP43-GFP in the nucleus or cytoplasm per cell (Fig. 6*F*). Collectively, these results demonstrated that RAN increases the nucleus import of TDP43 and acts as an adapter to enhance TDP43 binding to *G3BP1* mRNA, thereby increasing the G3BP1 expression level.

### RAN promotes AKT and ERK signaling *via* G3BP1 to facilitate NPC progression

**Figure 7:**
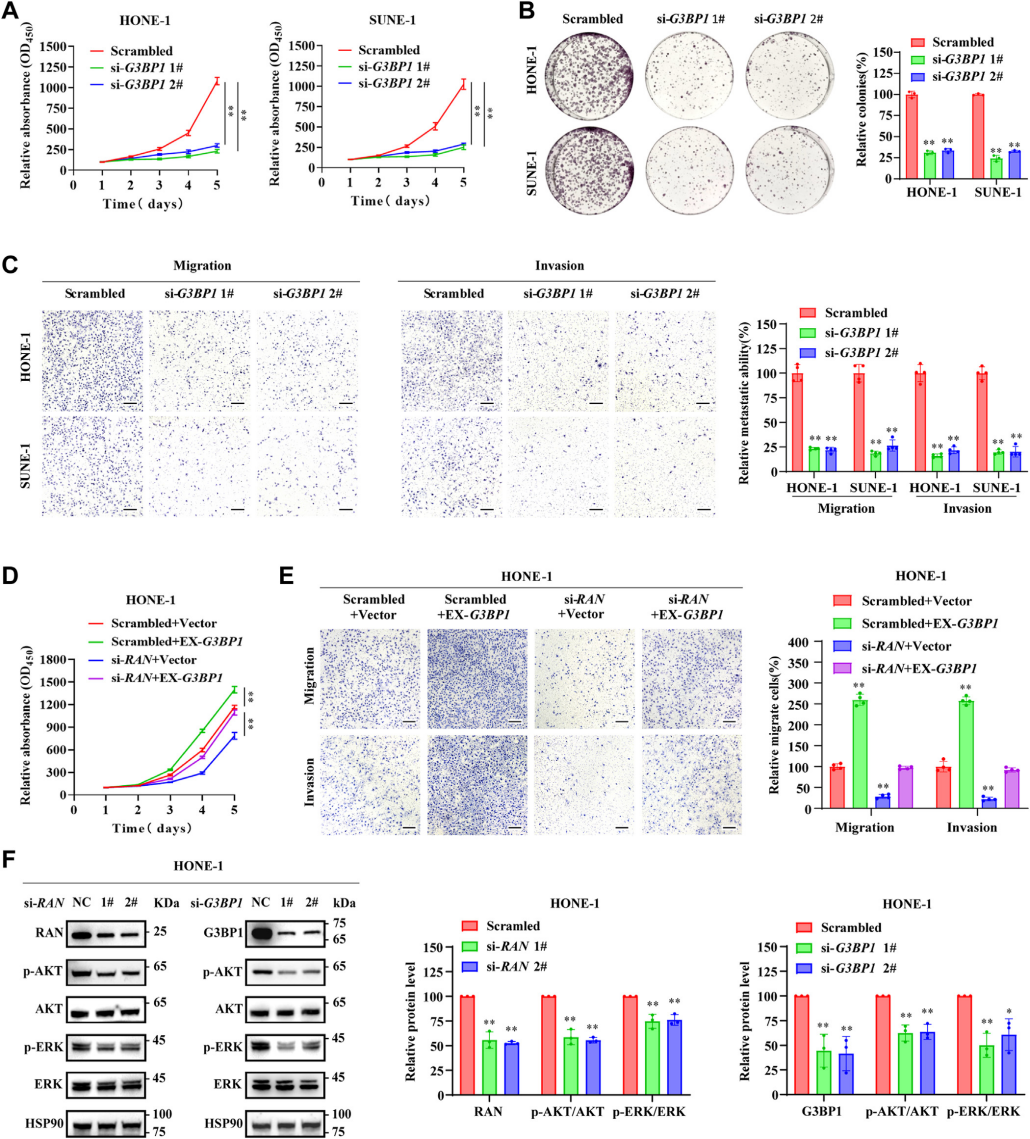
**RAN facilitates AKT/ERK phosphorylation *via* G3BP1 and further promotes NPC progression.***A* and *B,* cell proliferation ability was evaluated by CCK-8 assays in SUNE-1 and HONE-1 cells after silencing of G3BP1. Data are presented as the mean ± SD (n = 6). *B,* cell proliferation ability was evaluated by colony formation assays in SUNE-1 and HONE-1 cells after silencing of G3BP1. Data are presented as the mean ± SD (n = 3). *C,* migration and invasion capacity were analyzed by transwell assays in G3BP1-silenced SUNE-1 and HONE-1 cells. The scale bar represents 200 μm. Data are presented as the mean ± SD (n = 4). *D,* cell proliferation ability was evaluated by CCK-8 assays in HONE-1 cells after cotransfected with scrambled control or si-RAN, together with empty vector or HA-tagged G3BP1 overexpression vector. Data are presented as the mean ± SD (n = 6). *E,* migration and invasion capacity were analyzed by transwell assays in HONE-1 cells after cotransfected with scrambled control or si-RAN, together with empty vector or HA-tagged G3BP1 overexpression vector. Data are presented as the mean ± SD (n = 4). *F,* AKT, p-AKT, ERK, and p-ERK protein levels with knockdown of RAN or G3BP1 were detected by Western blotting in HONE-1 cells. The blots are the representation of three independent experiments. Data are presented as the mean ± SD (n = 3). ∗*p* < 0.05 and ∗∗*p* < 0.01. The significant differences were assessed using one-way ANOVA (*B*, *C*, and *F*) and two-way ANOVA (*A* and *D*). CCK-8, Cell Counting Kit-8; HA, hemagglutinin; IHC, immunohistochemistry; NPC, nasopharyngeal carcinoma.

To confirm RAN promotes NPC progression *via* G3BP1, we verified the biological function of G3BP1 in HONE-1 and SUNE-1 cells. G3BP1 was silenced using two individual siRNAs (si-*G3BP1* 1# and si-*G3BP1* 2#, Fig. S9*A*). Similar to that of RAN silencing, knockdown of G3BP1 impaired the proliferation ability and the migration and invasion capacity of NPC cells (Fig. 7, *A*–*C*). The *in vitro* functional rescue experiments were conducted by overexpressing HA-tagged G3BP1 in HONE-1 and SUNE-1 cells (Fig. S9*B*), with or without RAN silencing. Matching our expectations, CCK-8 assays showed that overexpression of G3BP1 rescued the impaired proliferation ability of RAN-silenced NPC cells (Fig. 7*D* and Fig. S9*C*), transwell assays showed that overexpression of G3BP1 rescued the suppressive effect of RAN silencing on the migration and invasion capacity of NPC cells (Fig. 7*E* and Fig. S9*D*).

We then explored the downstream signaling pathway of RAN–G3BP1 axis. AKT and MAPK signaling have been proven to play important roles in NPC proliferation and metastasis (33, 34), so we first detected the expression level of AKT and ERK after RAN or G3BP1 silencing in HONE-1 and SUNE-1 cells. RAN or G3BP1 silencing did not affect total AKT and ERK levels, but both significantly decreased the level of phosphorylated AKT and ERK (Fig. 7*F* and Fig. S10*A*), consistent with previous evidence that G3BP1 could promote PI3K/AKT activation (35). The rescue experiments were conducted by cotransfected with HA-tagged G3BP1 overexpression vector and si-RAN in HONE-1 and SUNE-1 cells. Western blotting showed that overexpression of G3BP1 rescued the decreased level of phosphorylated AKT and ERK in RAN-silenced NPC cells (Fig. S10*B*). Taken together, these results revealed that RAN promotes the phosphorylation of AKT and ERK *via* G3BP1, and ultimately facilitates NPC proliferation and metastasis.

## Discussion

**Figure 8:**
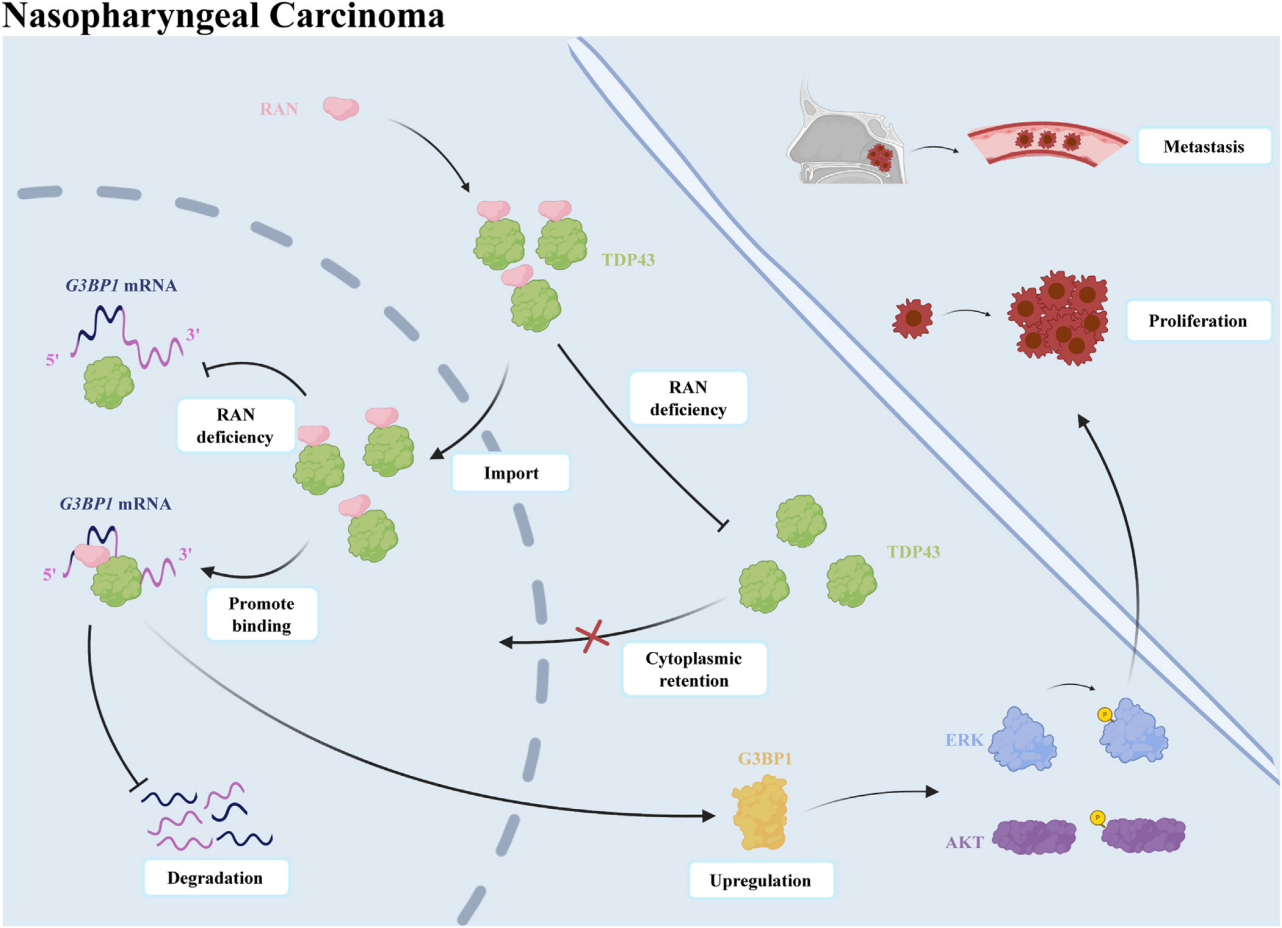
**Example diagram of RAN–TDP43–G3BP1 axis modulation.** RAN increases the nucleus import of TDP43 and acts as an adapter to facilitate TDP43 direct binding to *G3BP1* mRNA in the nucleus, thereby increasing *G3BP1* mRNA stability and leading to upregulation of G3BP1, which further enhances AKT and ERK signaling and ultimately facilitates NPC proliferation and metastasis. NPC, nasopharyngeal carcinoma.

In the present study, we elucidated that RAN is upregulated in NPC and can be performed as a prognostic biomarker for NPC patients. RAN increases the nucleus import of TDP43 and directly binds to the CDS of *G3BP1* mRNA to enhance TDP43 interaction with the 3′ UTR of *G3BP1* mRNA. These dual functions of RAN increase G3BP1 mRNA stability in the nucleus and lead to upregulation of G3BP1, further enhancing AKT and ERK signaling and ultimately facilitating NPC proliferation and metastasis (Fig. 8).

Except for RAN, the other four RBPs (EZH2, RDM1, HRSP12, and ALYREF) were also upregulated in NPC and promoted NPC cell migration or proliferation. The main function of EZH2 is trimethylating lysine 27 of histone H3 (H3K27me3) to silence gene transcription. But EZH2 also can act as an RBP to bind lncRNA and then be recruited to the promoters of downstream genes (36). RDM1 has a classical RNA binding domain called RNA recognition motif, but the downstream RNAs that can be bound and regulated by RDM1 remain unclear (37). HRSP12 can directly bind to m6A-containing RNAs and bridge interaction between YTHDF2 and RNase P/MRP, which promotes RNA degradation (38). ALYREF specifically binds to the m5C-modified RNAs and regulates their transportation between cytoplasm and nucleus (39). Moreover, these RBPs have been reported to be closely associated with various tumor proliferation, metastasis, immunity, and chemoresistance and can serve as prognostic markers (40, 41, 42, 43). However, their biological function and posttranscriptional regulation mechanism in NPC remain to be explored systematically. In future studies, we will also investigate whether RAN and the other four RBPs can serve as a gene signature to predict the survival of NPC patients.

RAN promotes tumor progression through a variety of mechanisms, and the classical one is regulating the nuclear import of critical tumor-promoting transcription factors and the cytoplasmic retention of tumor-suppressing transcription factors (31). For example, the silencing of RAN leads to a decreased nuclear localization of β-catenin and NF-κB, but an increased nuclear localization of p53 and p27, further inducing tumor cell apoptosis (44). As the research continues, more tumor-promoting mechanisms of RAN have been gradually discovered. Overexpression of LIN28B in neuroblastoma increases the levels of RAN by directly binding *RAN* mRNA, and then RAN induces phosphorylation of threonine 288 of AURKA and increases AURKA enzymatic activity, ultimately facilitating neuroblastoma progression (20). In ovarian cancer cells, RAN can interact with the RhoA protein dependent on its serine 188. After that, RAN can prevent the degradation of RhoA by impairing the proteasome pathway, and then facilitate the plasma membrane/ruffles distribution of RhoA. This special localization of RhoA is critical to ovarian cancer cell proliferation and invasion (21). A recent study has brought more attention to the ability of RAN to bind RNAs. RAN is involved in the nuclear export of circular RNAs, which is necessary for circular RNAs to perform their function in the cytoplasmic. Concretely, RAN is recruited to the circular RNA and directly binds to it after the interaction between this circular RNA and IGF2BP1. RAN can also act as an adapter to enhance the binding of IGF2BP1 to the circular RNA. Subsequently, RAN binds to exportin-2 and facilitates the nuclear export of circular RNA (26). Similarly, we found that RAN can not regulate mRNA nuclear export, but we suggested that RAN can directly bind and stabilize *G3BP1* mRNA in the nucleus. This finding has significance for refining the RAN’s posttranscriptional regulation mechanisms of downstream RNAs, but whether this regulatory mechanism can apply to other mRNAs needs to be further investigated.

G3BP1 is well known for its function in stress granule assembly and dynamics (45). Besides, G3BP1 can take part in Ras signal transduction *via* binding to the Ras-GTPase–activating protein, participate in the posttranscriptional regulation of RNA *via* its RNA binding ability, and control cellular senescence *via* the cyclic GMP-AMP synthase pathway (26, 46, 47). The multifunctional protein G3BP1 also has an important role in a variety of tumors which has been extensively investigated (48). The upregulation and the tumor-promoting function of G3BP1 in NPC were reported recently (17), but the factors increasing the level of G3BP1 in NPC remain elusive. TDP43 plays a crucial role in the amyotrophic lateral sclerosis and frontotemporal dementia disease spectrum (49). In the field of cancer, TDP43 also has an important function *via* directly binding RNA (29). TDP43 can bind to the UG-rich sequence in 3′ UTR of *ABHD2* mRNA and enhance its stability, further suppressing hepatocellular carcinoma cell apoptosis (50). TDP43 can regulate the alternative splicing of *CD44* pre-mRNA *via* binding to a UG repeat sequence between *CD44* pre-mRNA variant exon 10 and standard exon 6, ultimately facilitating the stemness of breast cancer stem cells (51). In our study, RAN acts as an adapter to facilitate direct binding of TDP43 to *G3BP1* mRNA 3′ UTR, thereby increasing *G3BP1* mRNA stability. Different from most of the RBPs regulating the mRNA stabilization in the cytoplasm (52, 53), we indicated that TDP43 stabilizes *G3BP1* mRNA in the nucleus, consistent with the evidence that the depletion of nuclear TDP43 is enough to suppress G3BP1 protein levels (54). Subsequent research has shown that RAN increases the nucleus import of TDP43 and enhances TDP43 nuclear distribution. Since TDP43 can also bind DNA (55), TDP43 nuclear depletion by RAN silencing might lead to aberrant expression of various genes in transcriptional or posttranscriptional ways, which can be focused on next research. Increasing AKT and ERK signaling has been observed in numerous types of tumors, including NPC. AKT and ERK signaling are key regulators in tumor progression and metastasis, which are crucial targets for tumor precision therapy (56, 57). Our study uncovered the mechanism for the increasing AKT and ERK signaling in NPC, which is helpful to the development of targeted medicine for NPC patients.

In conclusion, we discovered a new prognostic biomarker for NPC, high levels of RAN are associated with poor prognosis of NPC patients. This suggested that RBPs have great potential in the prediction of NPC prognosis. We found RAN facilitates NPC proliferation, migration, and invasion *in vitro* and *in vivo,* providing a possible therapeutic target of NPC. Furthermore, we showed that RAN is a noncanonical RBP that can bind and stabilize RNA. It expands the mechanism of RAN regulating tumor progression and emphasizes the importance of noncanonical RBPs in tumor regulation. We also discovered a new regulation model of RAN with dual functions. By recruiting TDP43 into the nucleus and enhancing its interaction with *G3BP1* mRNA, RAN can stabilize *G3BP1* mRNA in the nucleus. These suggested that RAN takes part in the posttranscriptional regulation of mRNA by combining its ability to regulate nucleo-cytoplasmic transportation of proteins and bind RNAs. In summary, our findings reveal a critical role of RAN in NPC progression and provide a new regulation framework for RBP-RNA.

## Experimental procedures

### Clinical specimens

This study was approved by the Institutional Ethical Review Board of Sun Yat-sen University Cancer Center (GZR2021-177). The study abided by the principles of the Declaration of Helsinki. A clinical cohort of NPC patients that were treated at Sun Yat-sen University Cancer Center (SYSUCC) between 2013 and 2014 and had formalin-fixed paraffin-embedded tumor samples stored at the Pathology Department of SYSUCC was established (N = 211). All patients received radical radiotherapy and had tumor biopsy before treatment initiation. The baseline characteristics of patients are shown in Table S5.

### Cells and culture conditions

All cell lines (HONE-1, SUNE-1, S26, S18, 6-10B, 5-8F, CNE-1, CNE-2, HNE-1, C666, HK-1, N2Bmil, and NP69) were authenticated and generously provided by Dr M. Zeng (Sun Yat-sen University Cancer Center) (58, 59, 60, 61, 62). Human NPC cells (HONE-1, SUNE-1, *etc.*) were cultured in Roswell Park Memorial Institute-1640 medium (Invitrogen) or Dulbecco's modified Eagle's medium (Invitrogen) based on the addition of 10% fetal bovine serum (ExCell Bio). Human immortalized nasopharyngeal epithelial cell lines (NP69 and N2Bmil) were cultured in keratinized cell serum-free medium (Invitrogen) containing bovine pituitary extract (BD Biosciences). HONE-1 and SUNE-1 cell lines were predominantly used because they derived from poorly differentiated NPC primary cultures (61, 62). All cell lines were stored in a humidified environment at 37 °C, 95% air, and 5% CO_2_. Cells were ensured to be passaged every 1 to 3 days during the experiment.

### Reverse transcription quantitative real-time PCR

Total RNA extraction from tissues or cells requires TRIzol Reagent (Ambion). Total RNA was reverse transcribed and synthesized into complementary DNA (cDNA) using HiScript III RT SuperMix (Vazyme) (63). The cDNA was then used as a template. The appropriate reagents were added to form a system for real-time fluorescence quantitative PCR (qPCR) detection using the CFX96 Touch and CFX384 Touch real-time PCR Detection System (Bio-Rad) or the LightCycler 480II (384 wells or 96 wells; Roche) and confirming that the Platinum SYBR Green qPCR SuperMix-UDG reagent (Vazyme) for real-time fluorescent qPCR detection. Threshold cycle counts were analyzed in triplicate for each sample. *GAPDH* or *U3* acted as an internal reference for cycle count value normalization. All the reactions were performed in triplicate. The primers required for this reaction are shown in Table S2.

### Western blotting

The cells to be studied were first lysed by adding radioimmunoprecipitation assay buffer (Millipore) containing protease and phosphatase inhibitors (Thermo Fisher Scientific) to the cells. Then the cell lysates were extracted to obtain total protein. Before Western blotting, the bicinchoninic acid Protein Assay Kit (Thermo Fisher Scientific) was used to determine the protein concentration. Then add 5 × loading buffer, mix, and boil at 90 °C to 100 °C for about 10 min. The sample was run at 10% homemade SDS polyacrylamide gel (Epizyme Biotech) or 4 to 20% preformed SDS-polyacrylamide gel (GeneScript) and transferred to polyvinylidene fluoride membranes (Millipore) (64). It was blocked with 5% skimmed milk or blocking solution and incubated overnight at 4 °C with the corresponding primary antibody. The membrane was washed three times with Tris-buffered saline with Tween 20 buffer and incubated with the corresponding secondary antibody for 1 h at room temperature. Finally, the membrane was washed three times with Tris-buffered saline with Tween 20 buffer. Imaging was performed on the appropriate luminometer. The results were quantified by gray-scale statistics using ImageJ (https://imagej.net/ij/). The antibodies used in this study are as follows: RAN (1:1000, Abcam, ab155103), TDP43 (1:1000, Abcam, ab190963), G3BP1 (1:2000, Proteintech, 13057-2-AP), eEF2 (1:2000, Proteintech, 20107-1-AP), GAPDH (1:5000, Abcam, ab181602), LaminB (1:1000, Proteintech, 12987-1-AP), phospho-AKT (1:2000, CST, #4060), AKT (1:2500, Beyotime, AF1777), phospho-ERK (1:1000, Beyotime, AF1891), ERK (1:2500, Beyotime, AF1051), α-tubulin (1:5000, Abcam, ab7291), HA-tag (1:5000, Abcam, ab9110), GST-tag (1:5000, Proteintech, 66001-2-Ig), and HSP90 (1:5000, Proteintech, 13171-1-AP).

### Plasmid construction, cell transfection, and lentiviral infection

When RNA was knocked down, duplex RNAi oligonucleotides targeting human *RAN, G3BP1, TDP43, EZH2, RDM1, HRSP12, ALYREF, SF1, RNASEH2A, C1QBP, NCBP2,* and *MAGOHB* mRNA sequences were first purchased synthetically from Suzhou GenePharma (Table S1). A scrambled duplex RNA oligonucleotide was used as an RNA-negative control. The designed specific shRNA against RAN or scrambled shRNA were cloned into pLKO.1 vector (Tsingke). See Table S3 for specific primers. For gene overexpression, cDNAs comprising the ORF of human RAN, TDP43, and G3BP1 with N-terminal HA-tag, as well as TDP43 with GFP-tag sequences were cloned into the pcDNA3.1(−) and pcDNA3.1(+). Cells were transiently transfected with Lipofectamine 3000 (Invitrogen) plasmid according to the manufacturer's instructions. To stably knock down *RAN*, lentiviruses expressing shRNA-targeting *RAN* or shRNA control were cotransfected with lentiviral packaging plasmids into the human embryonic kidney 293T cells. The lentivirus-containing supernatant was collected and filtered after 48 h of incubation. Follow the manufacturer's instructions to infect HONE-1 and SUNE-1 cells with the viral solution. The full-length, antisense, deletion fragment, and mutant fragment sequences of the G3BP1 transcript were synthesized by Tsingke Biotechnology and cloned into the pcDNA3.1(−) vector to generate plasmids for T7 transcription *in vitro*.

### Cell proliferation and colony formation assays

In the cell proliferation assays, the transfected cells were inoculated with 1 × 10^3^ cells per well in a 96-well plate, according to the production company's instructions. Cell viability was assayed using CCK-8 (APE x BIO) every 24 h for 5 days. In colony formation assays, transfected cells were inoculated with 1 × 10^3^ cells per well in a 6-well plate and cultured for about 7 days, when colonies could be detected, they were fixed in methanol and stained with hematoxylin. The results were analyzed using ImageJ software, a free and open-source software developed by the National Institutes of Health.

### Transwell migration and invasion assay

For the transwell migration and invasion assay, transfected cells were cultured at 5 × 10^4^ (migration assay) or 1 × 10^5^ (invasion assay) in 200 μl of serum-free medium. They were inoculated into the upper chamber for migration (without Matrigel) and invasion (with Matrigel) tests, respectively. Medium containing 10% fetal bovine serum was added to the lower chamber. Migration was incubated for 12 h and invasion was incubated for 16 h. Cells located on the lower surface of the upper chambers were fixed with methanol, stained with hematoxylin, and observed by an inverted microscope.

### RIP-seq and RIP-qPCR

Before performing the RIP assay, experiments were initiated according to the Magna RIPTM Kit (Millipore) following the manufacturer's protocol. Cell lysates were incubated with protein A/G magnetic beads coated with 5 μg of specific antibody or normal IgG overnight at 4 °C. The immunoprecipitated RNA was separated with elution buffer and purified for next-generation sequencing (RiboBio) or Quantitative real-time reverse transcription PCR. The primers required for the reaction are shown in the Table S4.

### Coomassie brilliant blue stain assay

The Coomassie brilliant blue stain assay was performed using the fast Coomassie brilliant blue stain kit (Beyotime), and experiments were initiated according to the manufacturer's instructions. After the gel electrophoresis, a suitable volume of Coomassie brilliant blue fast staining solution was added to gel according to its size. After 30 min of the staining, the gel was washed using ddH_2_O until the stained background was removed. The gel images were then photographed and saved.

### T7 transcription *in vitro*

Utilizing the Ribo RNAmax-T7 biotin-labeled transcription kit (RiboBio) according to the manufacturer's instructions. The preparation of linearized DNA template was carried out, the T7 *in vitro* transcription reaction system was constructed according to the instructions, 1 μl of DNase I was added to the reaction system to remove the DNA template, and then the purification system of RNA product was formulated according to the instructions. Finally, the resolubilization of the RNA product and the quality inspection were carried out to obtain the RNA probes.

### RNA pull-down assay

Biotin-labeled RNA pull-down probes were obtained with the Ribo RNAmax-T7 biotin-labeled transcription kit (RiboBio), According to the manufacturer's instructions, the biotin-labeled RNA probes were incubated with cell lysate at 4 °C overnight and pulled down by the action of streptavidin-coated magnetic beads (Invitrogen). The bound proteins were obtained and then analyzed by Western blotting or sent to LC/MS analysis (Win Innovate Bio). The MS results were obtained and analyzed. For a cell-free RNA pull-down experiment, purified proteins were purchased from TargetMOl: RAN-GST (*Escherichia coli* protein purification system, Human, TMPH-01419), TDP43 (*E. coli* protein purification system, Human, TMPH-02178), eEF2 (*E. coli* protein purification system, Human, TMPH-01286), and GST (Baculovirus Insect Cells protein purification system, Schistosoma japonicum, TMPY-02157). The biotin-labeled RNA probes were incubated with purified proteins (5 μg) at 4 °C overnight and pulled down by the action of streptavidin-coated magnetic beads (Invitrogen). The bound proteins were obtained and then analyzed by Western blotting.

### FISH and IF

The colocalization and interaction of *G3BP1* mRNA and RAN or TDP43 protein were detected by FISH and IF costaining. Cy3-labeled *G3BP1* FISH probes were purchased and synthesized by RiboBio. *G3BP1* FISH probes consist of multiple 21-nt ssDNA sequences of *G3BP1* which are labeled by Cy3. They can specifically recognize and bind to *G3BP1* mRNA. After the sample was fixed, permeated, and washed, the cells were incubated with *G3BP1* probes overnight and then washed with saline-sodium citrate buffer. Subsequently, the sample was blocked with the IF blocking solution and incubated with an anti-RAN antibody (1:100, Abcam, ab155103) or anti-TDP43 antibody (1:100, Abcam, ab190963). 4′, 6-diamino-2-phenylindole staining of the nucleus. Finally, the samples were scanned and analyzed using a confocal microscope (Carl Zeiss AG, LSM 980). The colocalization and interaction of RAN and TDP43 protein, and TDP43 protein distribution with or without RAN silencing were detected by IF staining. After the sample was fixed, permeated, and washed, the cells were incubated with an anti-RAN antibody (1:100, Abcam, ab155103) and an anti-TDP43 antibody (1:100, Abcam, ab190963). 4', 6-diamino-2-phenylindole staining of the nucleus. Finally, the samples were scanned and analyzed using a confocal microscope (Carl Zeiss AG, LSM 980). For radial line profile analysis, the fluorescence intensity of RAN, TDP43, and *G3BP1* mRNA was been assessed by line scans of captured images. The spatial distribution curves of fluorescence intensity were used to analyze the colocalization.

### Cytoplasmic and nuclear RNA purification assay

Isolation of cytoplasmic and nuclear RNA using cytoplasmic and nuclear RNA purification kit (NORGEN). The cultured cells, after washing, were lysed by adding 200 μl of ice-cold Lysis buffer, centrifuged, and the supernatant (containing cytoplasmic RNA) and precipitate (containing nuclear RNA) were retained. After that, the nuclear RNA from the precipitate and cytoplasmic RNA from the supernatant were purified separately. After that, the nuclear RNA from the precipitate and cytoplasmic RNA from the supernatant were purified separately. Cytoplasmic RNA and nuclear RNA were available for subsequent RT-qPCR.

### Cytoplasmic and nuclear protein extraction assay

Extraction of nuclear and cytoplasmic proteins from samples using the ProteinExt Mammalian Nuclear and Cytoplasmic Protein Extraction Kit (TransGen Biotech). CPEB I and CPEB II were added to the cells and incubated on ice with shaking and mixing. After centrifugation to collect the supernatant (cytoplasmic proteins), the precipitate was added to CPEB I. After centrifugation with shaking, the precipitate was added to nuclear protein extraction buffer, and incubated on ice with shaking. Centrifuge to collect the supernatant (nuclear proteins). The cytoplasmic and nuclear proteins final storage at −80 °C for subsequent experiments.

### Silver stain assay

The silver staining test was performed using the Fast Silver Stain Kit (Beyotime), and experiments were initiated according to the manufacturer's instructions. After electrophoresis, the gel is fixed in 100 ml of fixative, and silver stained by step. Stained bands were used to observe differential enrichment proteins.

### Measurement of RNA stability

RNA stability was detected by RNA decay assay. NPC cells were first inoculated in 6-well plates and cultured until fusion. Actinomycin D (HY-17559, MedChemExpress) was added to each well until the final concentration was 10 μg/ml. The abundance of G3BP1 mRNA and 18S rRNA (normalized to time 0) was detected by quantitative PCR. The control RNA was 18S rRNA because actinomycin treatment did not affect 18S rRNA levels.

### Coimmunoprecipitation

Co-IP assays were performed using the PierceTM Co-IP Kit (Thermo Fisher Scientific). Immunoprecipitation (IP) buffers containing protease and phosphatase inhibitors (Thermo Fisher Scientific) were added to lysate the cells and centrifugated to obtain the lysate supernatant. The lysate was incubated overnight with corresponding specific antibodies in mild rotation at 4 °C. Immunocomplexes were recovered and washed 10 times with IP buffer. The enriched proteins are used for Western blotting analysis or sent to LC/MS analysis (Win Innovate Bio). The MS results were obtained and analyzed. The antibodies used in this study are as follows: RAN (1:50, Abcam, ab155103), TDP43 (1:50, CST, #3448). For a cell-free co-IP experiment, 5 μg purified TDP43 protein was mixed with 5 μg purified RAN-GST or GST protein. Then, anti-GST magnetic beads (Beyotime) were added to the protein solution and incubated overnight in mild rotation at 4 °C. Immunocomplexes were recovered and washed 10 times with IP buffer. The enriched proteins are used for Western blotting analysis.

### *In vivo* tumor xenograft models

All animal experimental procedures were approved by the Institutional Animal Care and Use Committee of Sun Yat-sen University Cancer Center (L025501202305016). Female BALB/c nude mice of about 19 g at 6 weeks of age were purchased from Guangdong Vital River Lab Animal Technology. For establishing a subcutaneous xenograft model, 1 × 10^6^ SUNE-1 cells stably expressing scrambled or sh-*RAN* were inoculated subcutaneously into the axilla of nude mice. Tumor volumes were measured every 2 days. After 20 days of growth, mice were executed, and subcutaneous tumors were isolated. For the inguinal lymph node metastasis model, 2 × 10^5^ scrambled or sh*-RAN* SUNE-1 cells were injected into mouse foot pads. After 4 weeks of growth, mice were executed, and footpad tumors and inguinal lymph nodes were isolated. Subcutaneous and footpad tumors and lymph nodes were paraffin-embedded and subjected to immunohistochemical analysis.

### IHC staining

For immunohistochemical staining assays, after the pre-processed description in our previous study (65), the sections from NPC patients were incubated with RAN antibody (1:400, Proteintech, 10469-1-AP) or anti-pan-cytokeratin antibody (ZSGB-BIO) overnight at 4 °C. On the next day, these sections were incubated with the corresponding secondary antibody (ZSGB-BIO) at 37 °C for 30 min. IHC-stained sections were finally obtained after the DAB chromogenic reaction. IHC staining analysis was performed using the semiquantitative scoring criteria of the IRS system, which was previously described (66). The IRS system categorizes the intensity of staining as nonexistent (0), weak (1), moderate (2), or strong (3), and the percentage of cellular staining as unstained (0), less than 25% stained (1), 25 to 50% stained (2), 51 to 75% stained (3), or more than 75% stained (4). IRS was calculated by the multiplication of these two variables. NPC patients were categorized into two groups of low RAN levels (IRS 0∼6) and high RAN levels (IRS 7∼12) for statistical analysis.

### Statistical analyses

GraphPad Prism (version 9.0; GraphPad Inc), IBM SPSS (version 24.0; IBM Corp), and R (version 3.6) software for Windows were used for statistical analysis. Data are expressed as mean ± SD or mean ± 95% confidence interval. Continuous variables were compared using Student's *t* test (unpaired, two-tailed), and categorical variables were compared using the *chi*-square test. One-way ANOVA and two-way ANOVA followed by the Bonferroni test were used for multiple comparisons. The significant differences in correlations were assessed using the Pearson correlation analysis. Survival curves were assessed using the Kaplan–Meier method. The log-rank test was used to compare differences in survival outcomes. Single and multifactor Cox regression analyses were used to calculate hazard ratios and 95% confidence intervals. A *p* value of <0.05 was considered statistically significant. ∗, *p* < 0.05; ∗∗, *p* < 0.01.

## Data availability

RIP-seq and RNA-seq data are accessible at the GEO Repository Knowledge base (GSE262035). The MS proteomics data and detailed approaches have been deposited to the ProteomeXchange Consortium (https://proteomecentral.proteomexchange.org) *via* the iProX partner repository (67, 68) with the dataset identifier PXD056577. The GEO accession numbers referenced in the manuscript are GSE126683, GSE53819, GSE103611, GSE13597, GSE68799, and GSE102349. In addition to the RNA-seq data of NPC tumor tissue measured by our team (GSE126683), The others (GSE53819, GSE103611, GSE13597, GSE68799, and GSE102349) were taken from RNA-seq datasets of tumor tissue from NPC patients collected from different hospitals over the past 15 years. The data used and analyzed in this article, as well as the source data supporting the results of this study, are available from the corresponding author upon request.

## Supporting information

This article contains supporting information.

## Conflict of interest

The authors declare that they have no conflicts of interest with the contents of this article.

## References

[1] Van Nostrand E.L., Freese P., Pratt G.A., Wang X., Wei X., Xiao R. (2020). A large-scale binding and functional map of human RNA-binding proteins. Nature.

[2] Gerstberger S., Hafner M., Tuschl T. (2014). A census of human RNA-binding proteins. Nat. Rev. Genet..

[3] Qin H., Ni H., Liu Y., Yuan Y., Xi T., Li X. (2020). RNA-binding proteins in tumor progression. J. Hematol. Oncol..

[4] Zhao Y., Mir C., Garcia-Mayea Y., Paciucci R., Kondoh H., LLeonart M.E. (2022). RNA-binding proteins: underestimated contributors in tumorigenesis. Semin. Cancer Biol..

[5] Gebauer F., Schwarzl T., Valcárcel J., Hentze M.W. (2021). RNA-binding proteins in human genetic disease. Nat. Rev. Genet..

[6] Wang J., Wang F., Ke J., Li Z., Xu C., Yang Q. (2022). Inhibition of *METTL3* attenuates renal injury and inflammation by alleviating *TAB3* m6A modifications via IGF2BP2-dependent mechanisms. Sci. Transl. Med..

[7] Sun Y., Dai H., Dai X., Yin J., Cui Y., Liu X. (2023). m1A in CAG repeat RNA binds to TDP-43 and induces neurodegeneration. Nature.

[8] Mohibi S., Chen X., Zhang J. (2019). Cancer the‘RBP’eutics–RNA-binding proteins as therapeutic targets for cancer. Pharmacol. Ther..

[9] Wang S., Sun Z., Lei Z., Zhang H.-T. (2022). RNA-binding proteins and cancer metastasis. Semin. Cancer Biol..

[10] Xu Y., Huangyang P., Wang Y., Xue L., Devericks E., Nguyen H.G. (2021). ERα is an RNA-binding protein sustaining tumor cell survival and drug resistance. Cell.

[11] Zhou Y.-J., Yang M.-L., He X., Gu H.-Y., Ren J.-H., Cheng S.-T. (2024). RNA-binding protein RPS7 promotes hepatocellular carcinoma progression via LOXL2-dependent activation of ITGB1/FAK/SRC signaling. J. Exp. Clin. Cancer Res..

[12] Bray F., Ferlay J., Soerjomataram I., Siegel R.L., Torre L.A., Jemal A. (2018). Global cancer statistics 2018: GLOBOCAN estimates of incidence and mortality worldwide for 36 cancers in 185 countries. CA. Cancer J. Clin..

[13] Chen Y.-P., Chan A.T.C., Le Q.-T., Blanchard P., Sun Y., Ma J. (2019). Nasopharyngeal carcinoma. Lancet.

[14] Qiao H., Tan X.-R., Li H., Li J.-Y., Chen X.-Z., Li Y.-Q. (2022). Association of intratumoral microbiota with prognosis in patients with nasopharyngeal carcinoma from 2 hospitals in China. JAMA Oncol..

[15] Zhang L., Huang Y., Hong S., Yang Y., Yu G., Jia J. (2016). Gemcitabine plus cisplatin versus fluorouracil plus cisplatin in recurrent or metastatic nasopharyngeal carcinoma: a multicentre, randomised, open-label, phase 3 trial. Lancet.

[16] Xiang X., Liu Y., Kang Y., Lu X., Xu K. (2022). MEX3A promotes nasopharyngeal carcinoma progression via the miR-3163/SCIN axis by regulating NF-κB signaling pathway. Cell Death Dis..

[17] Zhan Y., Wang W., Wang H., Xu Y., Zhang Y., Ning Y. (2024). G3BP1 interact with JAK2 mRNA to promote the malignant progression of nasopharyngeal carcinoma via activating JAK2/STAT3 signaling pathway. Int. J. Biol. Sci..

[18] Zheng Z.-Q., Li Z.-X., Guan J.-L., Liu X., Li J.-Y., Chen Y. (2020). Long noncoding RNA TINCR-mediated regulation of acetyl-CoA metabolism promotes nasopharyngeal carcinoma progression and chemoresistance. Cancer Res..

[19] Boudhraa Z., Carmona E., Provencher D., Mes-Masson A.-M. (2020). Ran GTPase: a key player in tumor progression and metastasis. Front. Cell Dev. Biol..

[20] Schnepp R.W., Khurana P., Attiyeh E.F., Raman P., Chodosh S.E., Oldridge D.A. (2015). A LIN28B-RAN-AURKA signaling network promotes neuroblastoma tumorigenesis. Cancer Cell.

[21] Zaoui K., Boudhraa Z., Khalifé P., Carmona E., Provencher D., Mes-Masson A.-M. (2019). Ran promotes membrane targeting and stabilization of RhoA to orchestrate ovarian cancer cell invasion. Nat. Commun..

[22] El-Tanani M., Platt-Higgins A., Lee Y.-F., Al Khatib A.O., Haggag Y., Sutherland M. (2022). Matrix metalloproteinase 2 is a target of the RAN-GTP pathway and mediates migration, invasion and metastasis in human breast cancer. Life Sci..

[23] Baltz A.G., Munschauer M., Schwanhäusser B., Vasile A., Murakawa Y., Schueler M. (2012). The mRNA-bound proteome and its global occupancy profile on protein-coding transcripts. Mol. Cell..

[24] Castello A., Fischer B., Eichelbaum K., Horos R., Beckmann B.M., Strein C. (2012). Insights into RNA biology from an atlas of mammalian mRNA-binding proteins. Cell.

[25] Li Y., Zhao D.Y., Greenblatt J.F., Zhang Z. (2013). RIPSeeker: a statistical package for identifying protein-associated transcripts from RIP-seq experiments. Nucleic Acids Res..

[26] Ngo L.H., Bert A.G., Dredge B.K., Williams T., Murphy V., Li W. (2024). Nuclear export of circular RNA. Nature.

[27] Zhang C.-H., Wang J.-X., Cai M.-L., Shao R., Liu H., Zhao W.-L. (2019). The roles and mechanisms of G3BP1 in tumour promotion. J. Drug Target..

[28] Li W., Deng X., Chen J. (2022). RNA-binding proteins in regulating mRNA stability and translation: roles and mechanisms in cancer. Semin. Cancer Biol..

[29] Ma X., Ying Y., Xie H., Liu X., Wang X., Li J. (2021). The regulatory role of RNA metabolism regulator TDP-43 in human cancer. Front. Oncol..

[30] Milicevic N., Jenner L., Myasnikov A., Yusupov M., Yusupova G. (2024). mRNA reading frame maintenance during eukaryotic ribosome translocation. Nature.

[31] El-Tanani M., Nsairat H., Mishra V., Mishra Y., Aljabali A.A.A., Serrano-Aroca Á. (2023). Ran GTPase and its importance in cellular signaling and malignant phenotype. Int. J. Mol. Sci..

[32] Dos Passos P.M., Hemamali E.H., Mamede L.D., Hayes L.R., Ayala Y.M. (2024). RNA-mediated ribonucleoprotein assembly controls TDP-43 nuclear retention. PLoS Biol..

[33] Qi C.-L., Huang M.-L., Zou Y., Yang R., Jiang Y., Sheng J.-F. (2021). The IRF2/CENP-N/AKT signaling axis promotes proliferation, cell cycling and apoptosis resistance in nasopharyngeal carcinoma cells by increasing aerobic glycolysis. J. Exp. Clin. Cancer Res..

[34] Ding S., Gao Y., Lv D., Tao Y., Liu S., Chen C. (2022). DNTTIP1 promotes nasopharyngeal carcinoma metastasis via recruiting HDAC1 to DUSP2 promoter and activating ERK signaling pathway. EBioMedicine.

[35] Zheng X., Chen J., Deng M., Ning K., Peng Y., Liu Z. (2024). G3BP1 and SLU7 jointly promote immune evasion by downregulating MHC-I via PI3K/akt activation in bladder cancer. Adv. Sci..

[36] Mirzaei S., Gholami M.H., Hushmandi K., Hashemi F., Zabolian A., Canadas I. (2022). The long and short non-coding RNAs modulating EZH2 signaling in cancer. J. Hematol. Oncol.J Hematol. Oncol..

[37] Messaoudi L., Yang Y.-G., Kinomura A., Stavreva D.A., Yan G., Bortolin-Cavaillé M.-L. (2007). Subcellular distribution of human RDM1 protein isoforms and their nucleolar accumulation in response to heat shock and proteotoxic stress. Nucleic Acids Res..

[38] Park O.H., Ha H., Lee Y., Boo S.H., Kwon D.H., Song H.K. (2019). Endoribonucleolytic cleavage of m6A-containing RNAs by RNase P/MRP complex. Mol. Cell..

[39] Zhang Q., Liu F., Chen W., Miao H., Liang H., Liao Z. (2021). The role of RNA m ^5^ C modification in cancer metastasis. Int. J. Biol. Sci..

[40] Wang B., Liu Y., Liao Z., Wu H., Zhang B., Zhang L. (2023). EZH2 in hepatocellular carcinoma: progression, immunity, and potential targeting therapies. Exp. Hematol. Oncol..

[41] Sheng J., Liu K., Sun D., Nie P., Mu Z., Chen H. (2021). Association of RDM1 with osteosarcoma progression via cell cycle and MEK/ERK signalling pathway regulation. J. Cell. Mol. Med..

[42] Yu J., Li W., Hou G., Sun D., Yang Y., Yuan S. (2023). Circular RNA cFAM210A, degradable by HBx, inhibits HCC tumorigenesis by suppressing YBX1 transactivation. Exp. Mol. Med..

[43] Yang Q., Wang M., Xu J., Yu D., Li Y., Chen Y. (2023). LINC02159 promotes non-small cell lung cancer progression via ALYREF/YAP1 signaling. Mol. Cancer.

[44] Yuen H.-F., Chan K.-K., Grills C., Murray J.T., Platt-Higgins A., Eldin O.S. (2012). Ran is a potential therapeutic target for cancer cells with molecular changes associated with activation of the PI3K/Akt/mTORC1 and Ras/MEK/ERK pathways. Clin. Cancer Res..

[45] Yang P., Mathieu C., Kolaitis R.-M., Zhang P., Messing J., Yurtsever U. (2020). G3BP1 is a tunable switch that triggers phase separation to assemble stress granules. Cell..

[46] Mao C., Wang X., Liu Y., Wang M., Yan B., Jiang Y. (2018). A G3BP1-interacting lncRNA promotes ferroptosis and apoptosis in cancer via nuclear sequestration of p53. Cancer Res..

[47] Omer A., Barrera M.C., Moran J.L., Lian X.J., Di Marco S., Beausejour C. (2020). G3BP1 controls the senescence-associated secretome and its impact on cancer progression. Nat. Commun..

[48] Ge Y., Jin J., Li J., Ye M., Jin X. (2022). The roles of G3BP1 in human diseases (review). Gene.

[49] Pasetto L., Grassano M., Pozzi S., Luotti S., Sammali E., Migazzi A. (2021). Defective cyclophilin A induces TDP-43 proteinopathy: implications for amyotrophic lateral sclerosis and frontotemporal dementia. Brain.

[50] Liu B., Wang X., Cao J., Chen L., Wang Y., Zhao B. (2022). TDP-43 upregulates lipid metabolism modulator ABHD2 to suppress apoptosis in hepatocellular carcinoma. Commun. Biol..

[51] Guo L., Ke H., Zhang H., Zou L., Yang Q., Lu X. (2022). TDP43 promotes stemness of breast cancer stem cells through CD44 variant splicing isoforms. Cell Death Dis..

[52] Wang X., Lu Z., Gomez A., Hon G.C., Yue Y., Han D. (2014). N6-methyladenosine-dependent regulation of messenger RNA stability. Nature.

[53] Finan J.M., Sutton T.L., Dixon D.A., Brody J.R. (2023). Targeting the RNA-binding protein HuR in cancer. Cancer Res..

[54] Sidibé H., Khalfallah Y., Xiao S., Gómez N.B., Fakim H., Tank E.M.H. (2021). TDP-43 stabilizes *G3BP1* mRNA: relevance to amyotrophic lateral sclerosis/frontotemporal dementia. Brain.

[55] Mackenzie I.R., Rademakers R., Neumann M. (2010). TDP-43 and FUS in amyotrophic lateral sclerosis and frontotemporal dementia. Lancet Neurol..

[56] Hua H., Zhang H., Chen J., Wang J., Liu J., Jiang Y. (2021). Targeting Akt in cancer for precision therapy. J. Hematol. Oncol.J Hematol. Oncol..

[57] Ullah R., Yin Q., Snell A.H., Wan L. (2022). RAF-MEK-ERK pathway in cancer evolution and treatment. Semin. Cancer Biol..

[58] Li X.-J., Peng L.-X., Shao J.-Y., Lu W.-H., Zhang J.-X., Chen S. (2012). As an independent unfavorable prognostic factor, IL-8 promotes metastasis of nasopharyngeal carcinoma through induction of epithelial-mesenchymal transition and activation of AKT signaling. Carcinogenesis.

[59] Lo A.K.F., Liu Y., Wang X.H., Huang D.P., Yuen P.W., Wong Y.C. (2003). Alterations of biologic properties and gene expression in nasopharyngeal epithelial cells by the Epstein-Barr virus-encoded latent membrane protein 1. Lab. Investig. J. Tech. Methods Pathol..

[60] Song L.-B., Li J., Liao W.-T., Feng Y., Yu C.-P., Hu L.-J. (2009). The polycomb group protein Bmi-1 represses the tumor suppressor PTEN and induces epithelial-mesenchymal transition in human nasopharyngeal epithelial cells. J. Clin. Invest..

[61] Yao K.T., Zhang H.Y., Zhu H.C., Wang F.X., Li G.Y., Wen D.S. (1990). Establishment and characterization of two epithelial tumor cell lines (HNE-1 and HONE-1) latently infected with Epstein-Barr virus and derived from nasopharyngeal carcinomas. Int. J. Cancer.

[62] Song L.-B., Zeng M.-S., Liao W.-T., Zhang L., Mo H.-Y., Liu W.-L. (2006). Bmi-1 is a novel molecular marker of nasopharyngeal carcinoma progression and immortalizes primary human nasopharyngeal epithelial cells. Cancer Res..

[63] Li J.-Y., Zhao Y., Gong S., Wang M.-M., Liu X., He Q.-M. (2023). TRIM21 inhibits irradiation-induced mitochondrial DNA release and impairs antitumour immunity in nasopharyngeal carcinoma tumour models. Nat. Commun..

[64] Chen Y., Zhao Y., Yang X., Ren X., Huang S., Gong S. (2022). USP44 regulates irradiation-induced DNA double-strand break repair and suppresses tumorigenesis in nasopharyngeal carcinoma. Nat. Commun..

[65] Li Z.-X., Zheng Z.-Q., Yang P.-Y., Lin L., Zhou G.-Q., Lv J.-W. (2022). WTAP-mediated m6A modification of lncRNA DIAPH1-AS1 enhances its stability to facilitate nasopharyngeal carcinoma growth and metastasis. Cell Death Differ..

[66] Weichert W., Denkert C., Schmidt M., Gekeler V., Wolf G., Köbel M. (2004). Polo-like kinase isoform expression is a prognostic factor in ovarian carcinoma. Br. J. Cancer.

[67] Ma J., Chen T., Wu S., Yang C., Bai M., Shu K. (2019). iProX: an integrated proteome resource. Nucleic Acids Res..

[68] Chen T., Ma J., Liu Y., Chen Z., Xiao N., Lu Y. (2022). iProX in 2021: connecting proteomics data sharing with big data. Nucleic Acids Res..

