# Supplementary material for: The noncanonical RNA-binding protein RAN stabilizes the mRNA of intranuclear stress granule assembly factor G3BP1 in nasopharyngeal carcinoma
Source: J Biol Chem. 2024 Nov 5;300(12):107964. doi: 10.1016/j.jbc.2024.107964 (PMC11635782; doi:10.1016/j.jbc.2024.107964)
Supplement: Supplemental Figures S1–S10 [file mmc1.docx]

**Supplementary Figures S1-S10**

**Supplementary Figure S1**

**
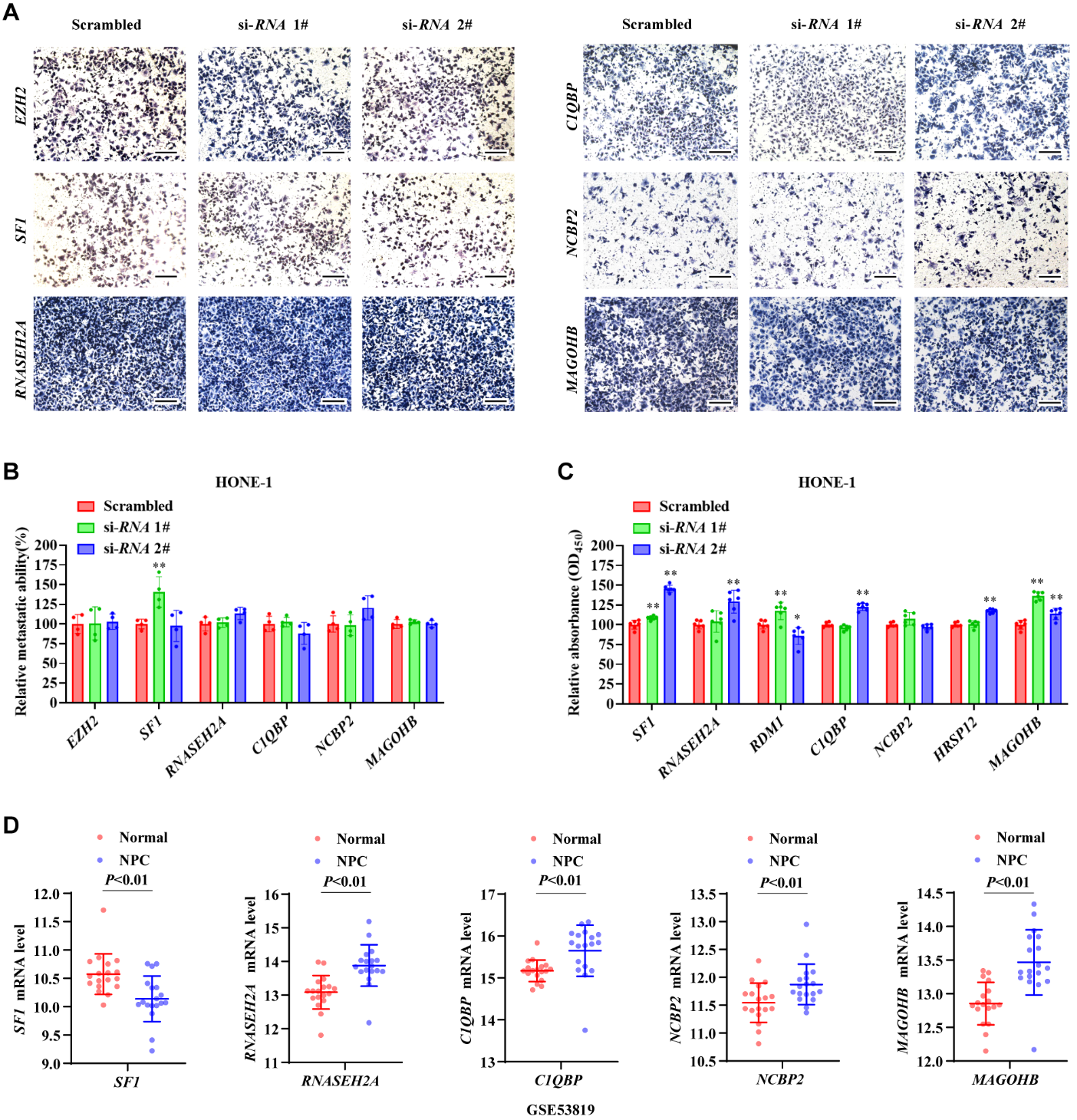
**

**Supplementary Figure S1. Search for RBPs affecting proliferation or metastasis in NPC.** A-B, Representative images(A) and quantified results(B) of the transwell migration assays in HONE-1 cells. Scale bar: 200 μm. Data are presented as the mean ± SD (n=4). C, identification of cell proliferation ability by CCK-8 assays after 96h of siRNA interference. Data are presented as the mean ± SD (n=6). D, Validation of *SF1*, *RNASEH2A*, *C1QBP*, *NCBP2*, and *MAGOHB* expression levels in NPC tissues (n=18) and normal nasopharyngeal tissues (n=18) based on data from GEO database GSE53819. Data are presented as the mean ± SD (n=18). **P* < 0.05, ***P* < 0.01. The significant differences were assessed using one-way ANOVA (B, C) and *t*-test (D).

**Supplementary Figure S2**


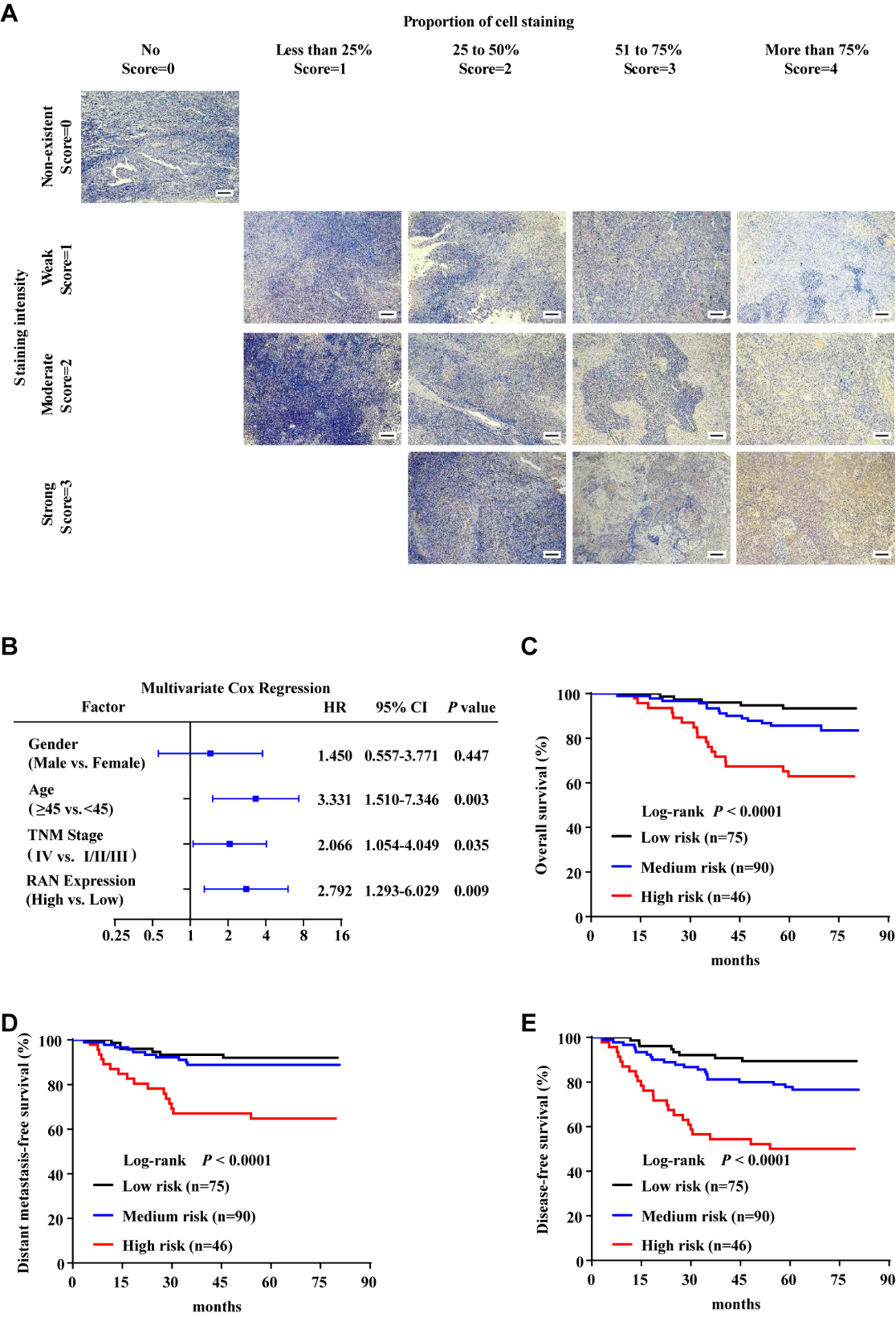


**Supplementary Figure S2. The prognostic value of RAN expression levels in NPC.** A, RAN expression levels were identified using the immunoreactivity score (IRS) system. Representative images of different immunoreactivity scores were shown. B, Multivariable Cox analysis of clinical prognostic parameters for overall survival of patients with NPC (n = 188). The hazard ratio (HR), 95% confidence interval (95% CI), and *P*-value are presented in the table. C-E, Kaplan–Meier curves of overall survival (C), distant metastasis-free survival (D), and disease-free survival (E) according to different risk grade, low risk (RAN low levels and Ⅰ-III TNM stage, n = 75), medium risk (RAN high levels or IV TNM stage, n = 90), and high risk (RAN high levels and IV TNM stage, n = 46). The log-rank test was used to compare differences in survival outcomes.

**Supplementary Figure S3**


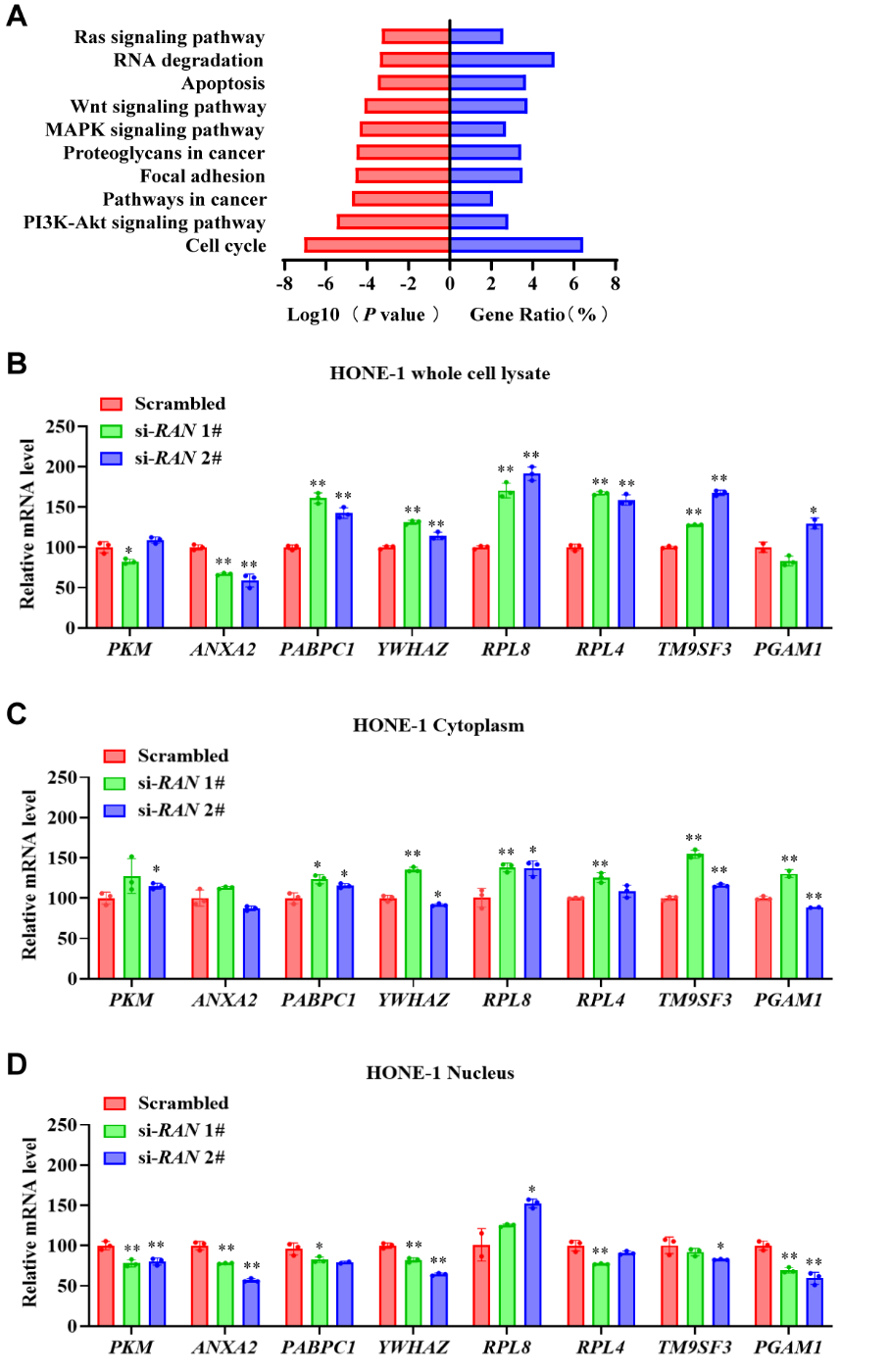


**Supplementary Figure S3. RAN knockdown did not lead to nuclear retention of gene transcripts.** A, KEGG pathway analysis of overlapping genes in RIP-seq from HONE-1 and SUNE-1 NPC cell lines. B-D, the top 8 genes identified by RIP-seq in HONE-1 and SUNE-1 cells were first analyzed, relative levels of these genes in whole cell lysate(A), cytoplasm(B), or nucleus(C), which normalized to *GAPDH* or *U3*, was indicated by RT-qPCR with or without RAN knockdown in HONE-1 cells. Data are presented as the mean ± SD (n=3). **P* < 0.05, ***P* < 0.01. The significant differences were assessed using one-way ANOVA (B-D).

**Supplementary Figure S4**


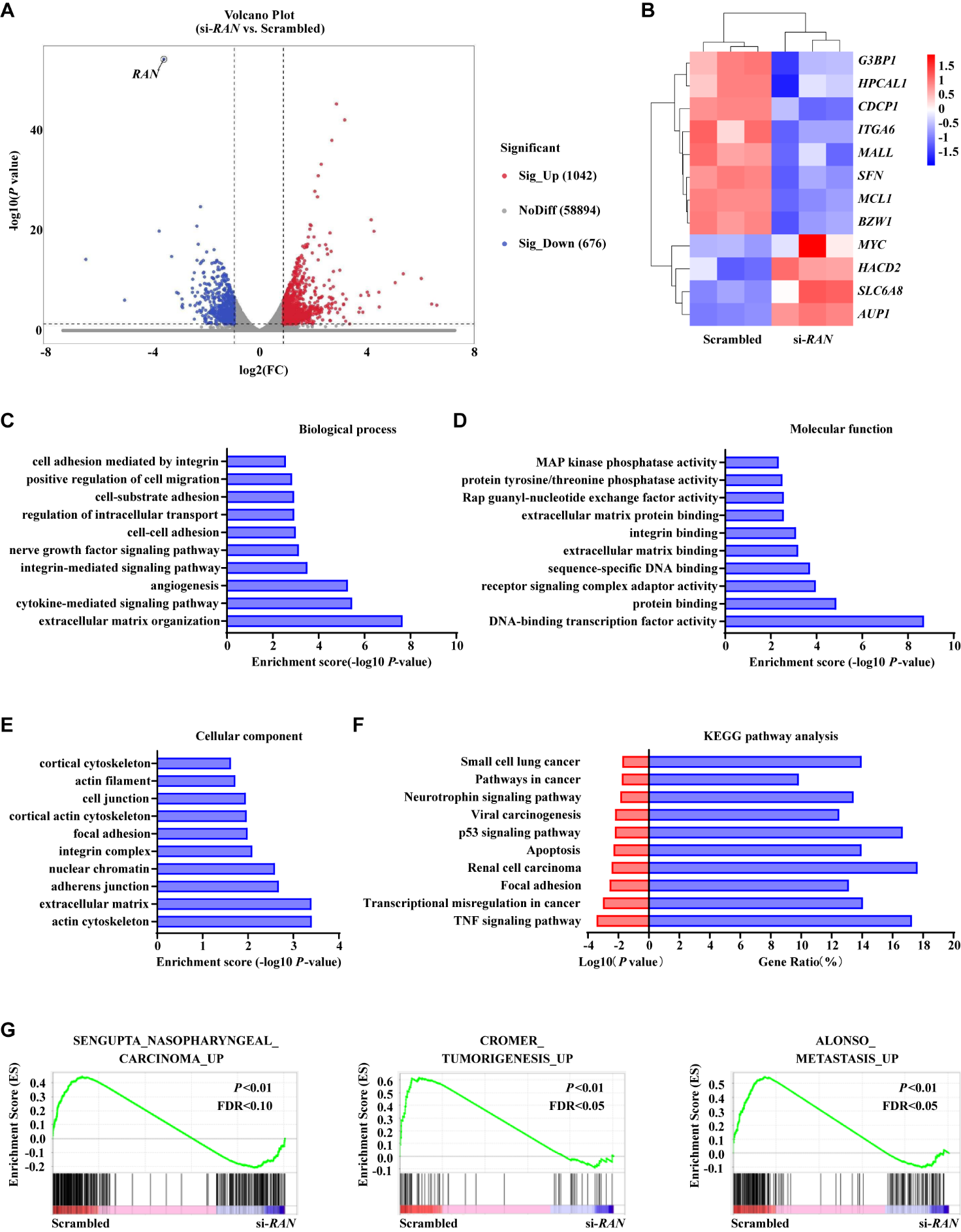


**Supplementary Figure S4. RNA-seq indicated RAN could promote NPC progression and metastasis.** A-G, the data from RNA-seq upon RAN knockdown was used for bioinformatics analysis. A, the Volcano Plot of differentially expressed genes after RAN silencing (|log2(fold change (FC))| >1 and *P* < 0.05). B, the heat map of the 12 overlapping genes in RNA-seq and RIP-seq. C-E, the Gene Oncology (GO) analysis of biological processes (C), molecular functions (D), and cellular components (E) of differentially expressed genes. F, Kyoto Encyclopedia of Genes and Genomes (KEGG) pathway analysis of differentially expressed genes. G, Gene Set Enrichment Analysis (GSEA) using data from the RNA-seq found that *RAN* expression levels were positively correlated with NPC tumorigenesis and metastasis.

**Supplementary Figure S5**

*
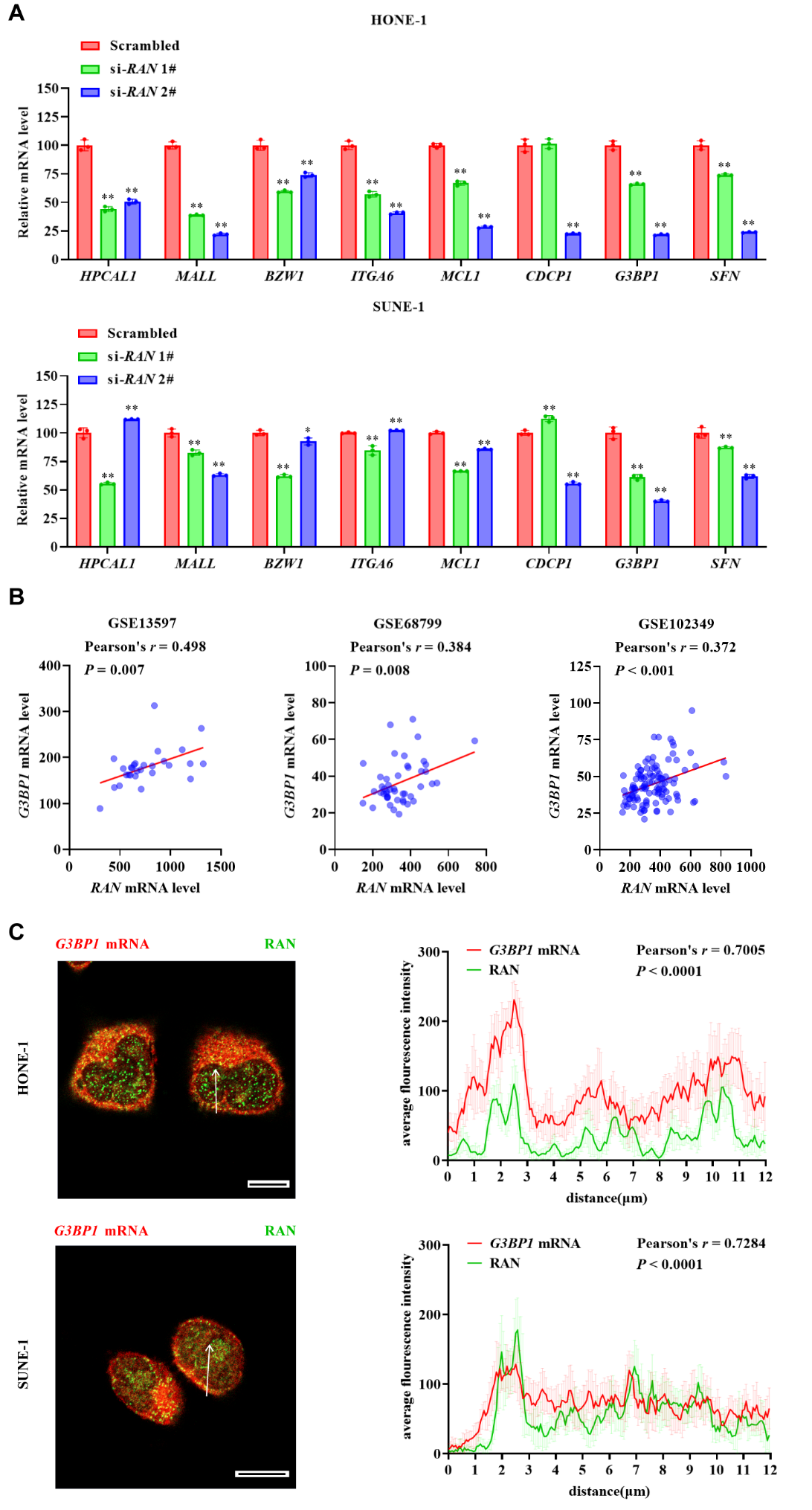
*

**Supplementary Figure S5. The levels of *G3BP1* are positively correlated with RAN.** A, relative levels of the 8 overlapping genes down-regulated after RAN silencing in RNA-seq were confirmed by RT-qPCR. Data are presented as the mean ± SD (n=3). B, Pearson correlation analysis of *RAN* and *G3BP1* levels in different GEO databases (GSE13597, GSE68799, and GSE102349). C, Radial line profile analysis showed the fluorescence intensity distribution of *G3BP1* mRNA and RAN protein in HONE-1 and SUNE-1 cells. **P* < 0.05, ***P* < 0.01. The significant differences were assessed using one-way ANOVA (A). The significant differences in correlations were assessed using the Pearson correlation analysis (B, C).

**Supplementary Figure S6**


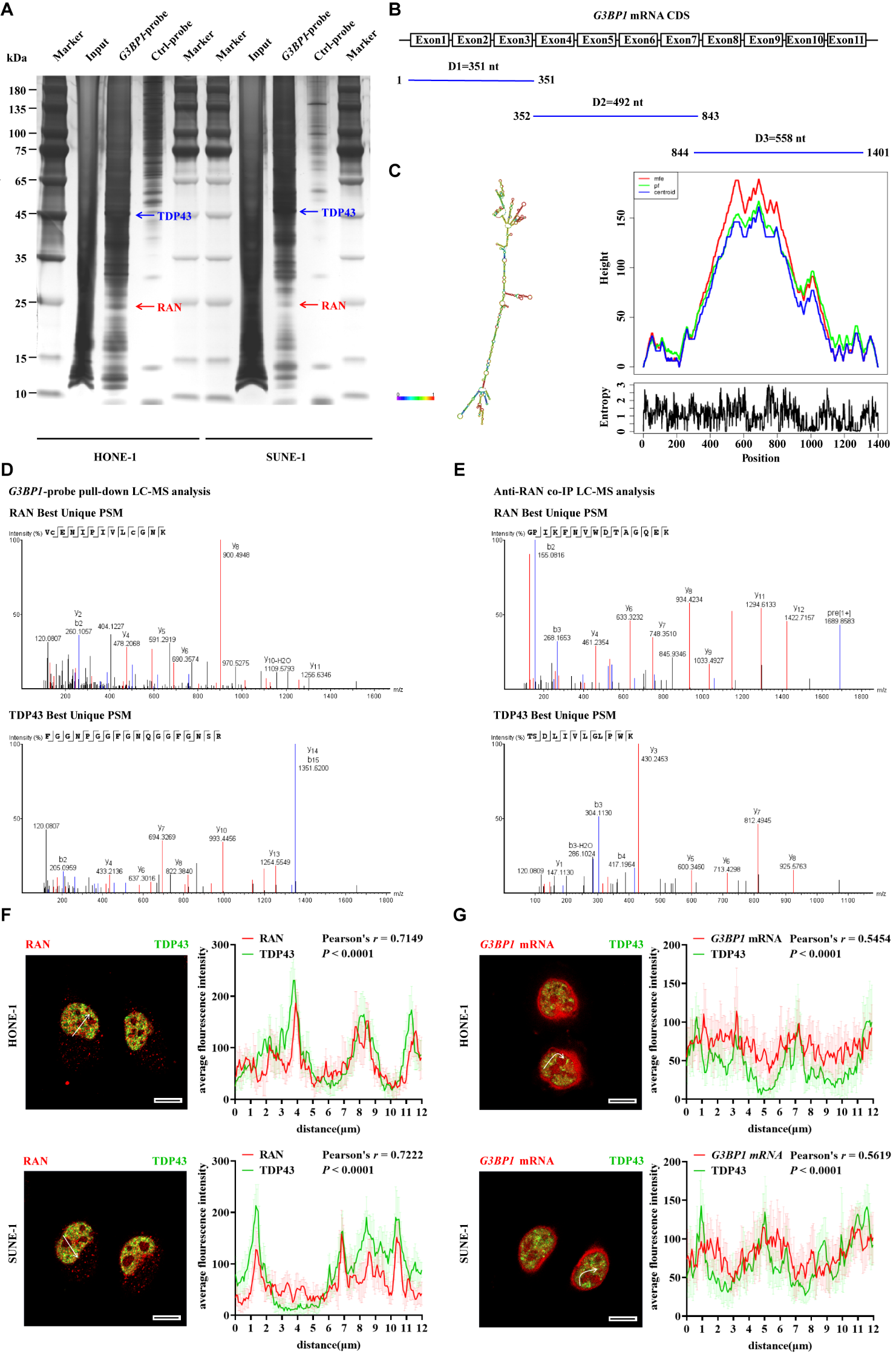


**Supplementary Figure S6. RAN, TDP43, and *G3BP1* mRNA form a complex in the nucleus.** A, RNA pulldown assays were conducted using biotin-labeled *G3BP1* probes from in vitro transcription or control antisense probes. The enriched proteins were followed by silver staining. B-C, construction of *G3BP1* mRNA deletion fragments based on exons(B) and online tool RNAfold WebServer(C). D, the enriched proteins pulled down by biotin-labeled *G3BP1* probes from in vitro transcription were subjected to mass spectrometry (MS) analysis. RAN and TDP43's best unique peptide-spectrum matches（PSM）were identified by MS analysis. E, the enriched proteins pulled down by anti-RAN antibody were subjected to mass spectrometry (MS) analysis. RAN and TDP43's best unique peptide-spectrum matches（PSM）were identified by MS analysis. F, Radial line profile analysis showed the fluorescence intensity distribution of RAN and TDP43 in HONE-1 and SUNE-1 cells. G, Radial line profile analysis showed the fluorescence intensity distribution of *G3BP1* mRNA and TDP43 protein in HONE-1 and SUNE-1 cells. The significant differences in correlations were assessed using the Pearson correlation analysis (F, G).

**Supplementary Figure S7**


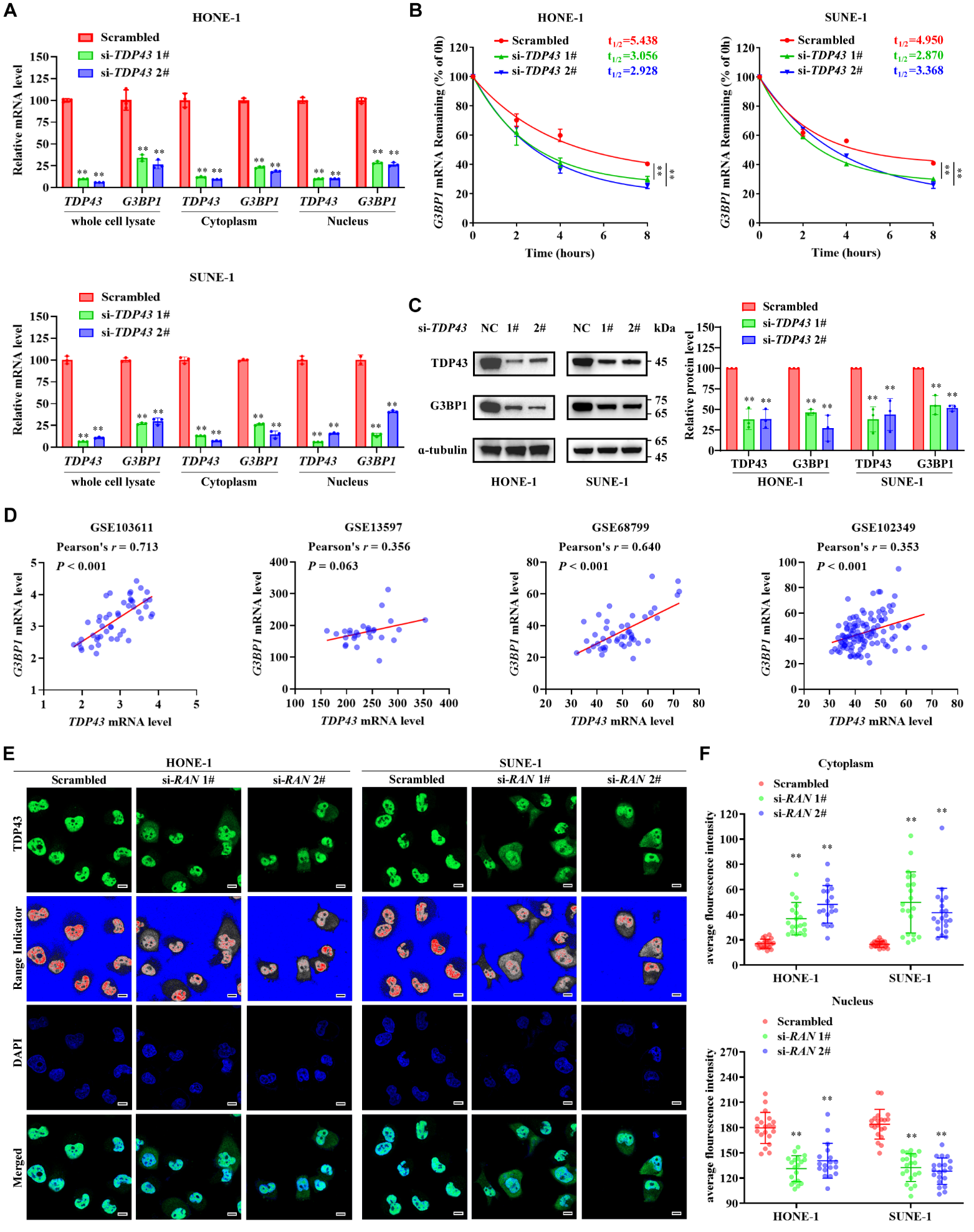


**Supplementary Figure S7. RAN stabilizes *G3BP1* mRNA via TDP43.** A, relative levels of G3BP1 mRNA levels in whole cell lysate, cytoplasm, or nucleus, which was normalized to GAPDH or U3, was indicated by RT-qPCR upon knockdown of TDP43 in HONE-1 and SUNE-1 cells. Data are presented as the mean ± SD (n=3). B, after treatment with actinomycin D (10 μg/ml), G3BP1 mRNA levels were quantified at indicated times in control and RAN-silenced cells. The half-life of G3BP1 mRNA was analyzed by plotting degradation curves. Data are presented as the mean ± 95%CI (n=3). C, G3BP1 protein levels with knockdown of TDP43 were detected by western blotting. The blots are the representation of three independent experiments. Data are presented as the mean ± SD (n=3). D, Pearson correlation analysis of *TDP43* and *G3BP1* levels in different GEO databases (GSE103611, GSE13597, GSE68799, and GSE102349). E, respectively recognizing TDP43 protein distribution (Green) in cells by IF with or without RAN silencing in HONE-1 and SUNE-1 cells, range indicator was performed to show clearer distribution of TDP43 in the nucleus or cytoplasm, cell nuclei were stained with DAPI (Blue). Scale bar: 10 μm. F, the average fluorescence intensity of TDP43 in the nucleus (lower) and cytoplasm (upper) of each cell was calculated. Data are presented as the mean ± SD (n=20). **P* < 0.05, ***P* < 0.01. The significant differences were assessed using one-way ANOVA (A, C, E) and two-way ANOVA (B). The significant differences in correlations were assessed using the Pearson correlation analysis (D).

**Supplementary Figure S8**


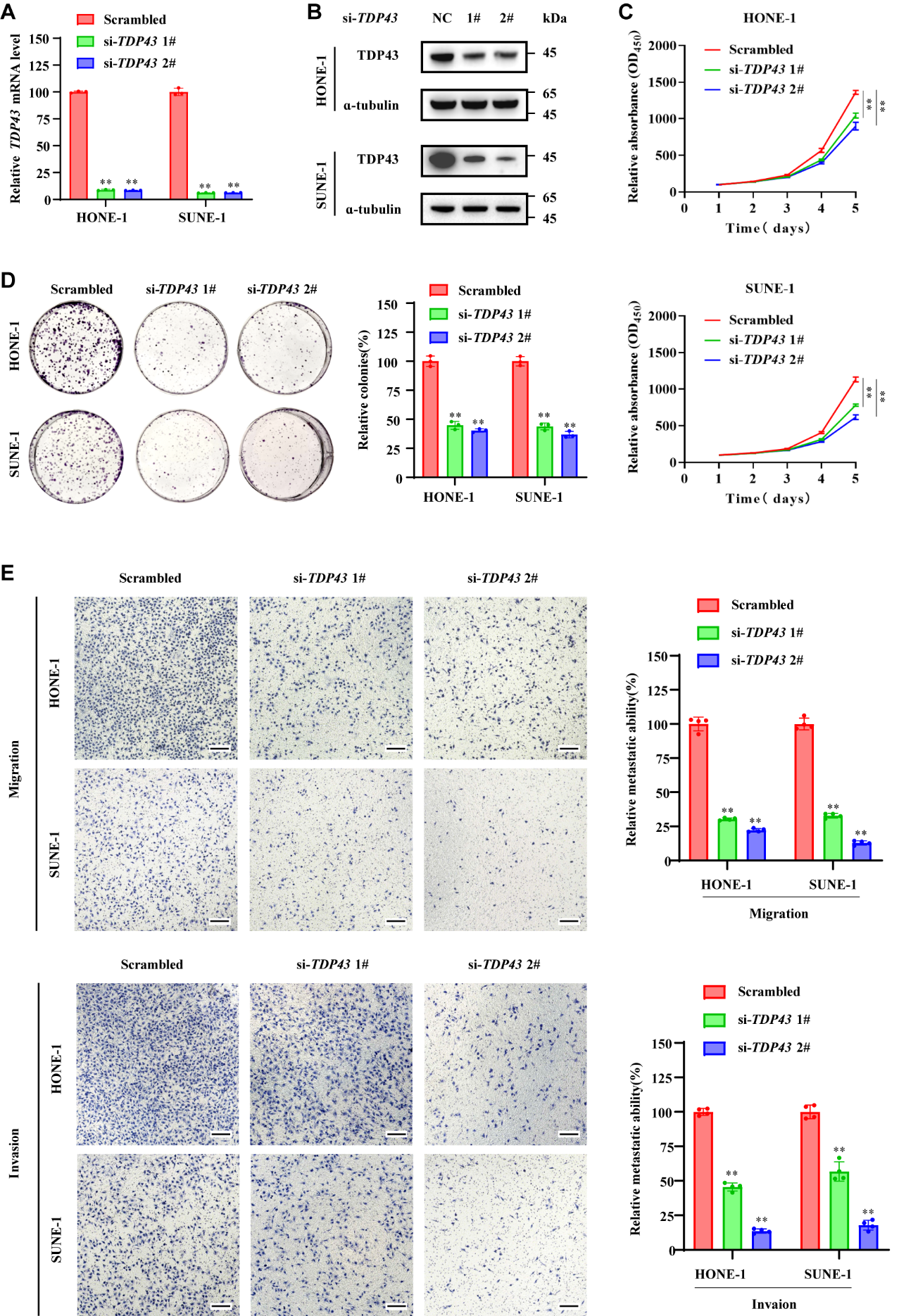


**Supplementary Figure S8. Silencing TDP43 impaired NPC cell proliferation, migration, and invasion *in vitro.*** A-B, siRNA interference was used to knockdown TDP43 in HONE-1 and SUNE-1 cells. RT-qPCR(A) and Western blotting(B) were used to verify knockdown efficiency. Data are presented as the mean ± SD (n=3). C, cell proliferation ability was evaluated by CCK-8 assay in SUNE-1 and HONE-1 cells after silencing of TDP43. Data are presented as the mean ± SD (n=6) D, cell proliferation ability was evaluated by colony formation assays in SUNE-1 and HONE-1 cells after silencing of TDP43. Data are presented as the mean ± SD (n=3). E, migration and invasion capacity were analyzed by transwell assays in TDP43-silenced SUNE-1 and HONE-1 cells. Scale bar: 200 μm. Data are presented as the mean ± SD (n=4). **P* < 0.05, ***P* < 0.01. The significant differences were assessed using one-way ANOVA (A, D, E) and two-way ANOVA (C).

**Supplementary Figure S9**

**
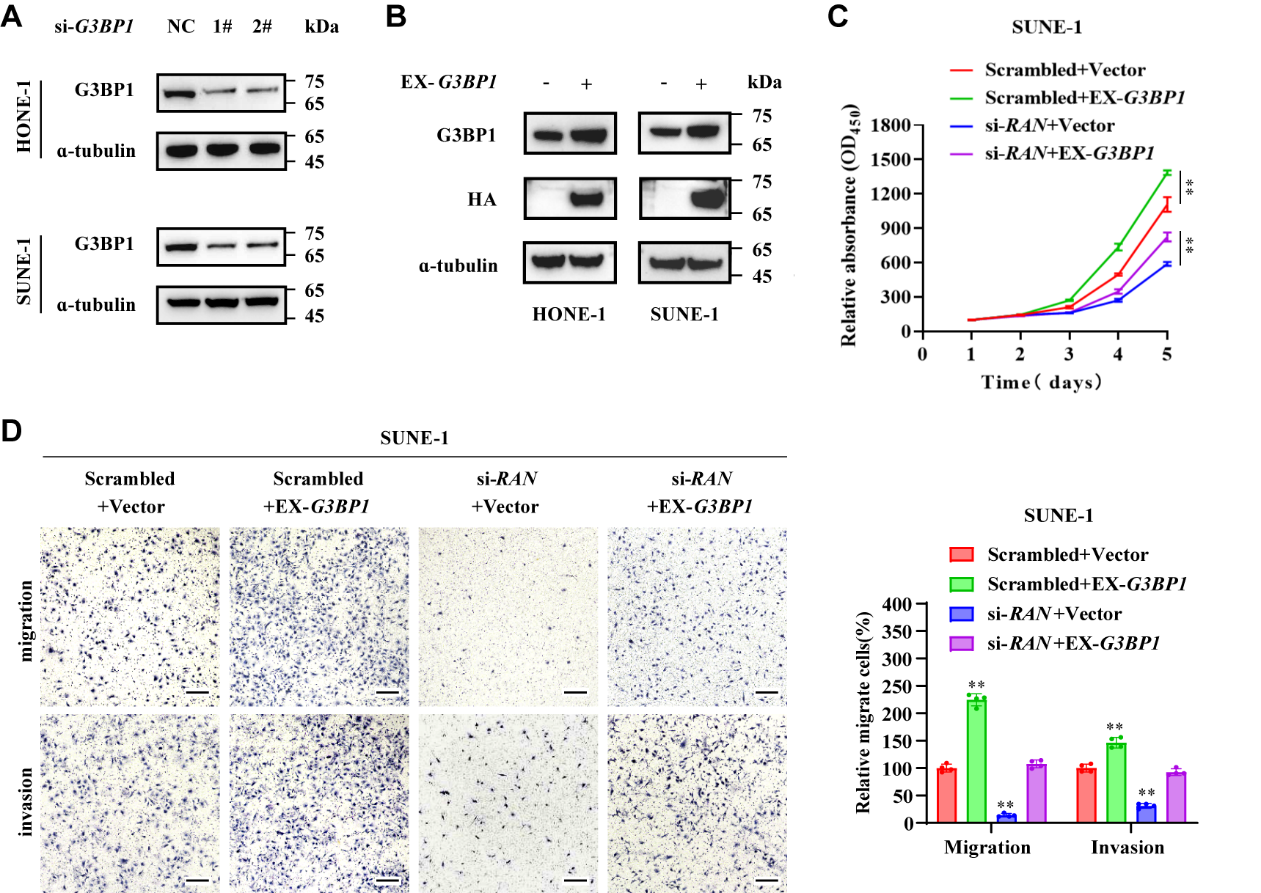
**

**Supplementary Figure S9.** **RAN facilitates** **NPC progression via G3BP1.** A, siRNA interference was used to knockdown G3BP1 in HONE-1 and SUNE-1 cells, and Western blotting was used to detect knockdown efficiency. B, Western blotting was used to confirm the transfection efficiency of the empty vector or HA-tagged G3BP1 overexpression vector in HONE-1 and SUNE-1 cells. C, cell proliferation ability was evaluated by CCK-8 assays in SUNE-1 cells after co-transfected with scrambled control or si-RAN, together with empty vector or G3BP1 overexpression vector. Data are presented as the mean ± SD (n=6). E, migration and invasion capacity were analyzed by transwell assays in SUNE-1 cells after co-transfected with scrambled control or si-RAN, together with empty vector or G3BP1 overexpression vector. Scale bar: 200 μm. Data are presented as the mean ± SD (n=4). **P* < 0.05, ***P* < 0.01. The significant differences were assessed using one-way ANOVA (D) and two-way ANOVA (C).

**Supplementary Figure S10**

**
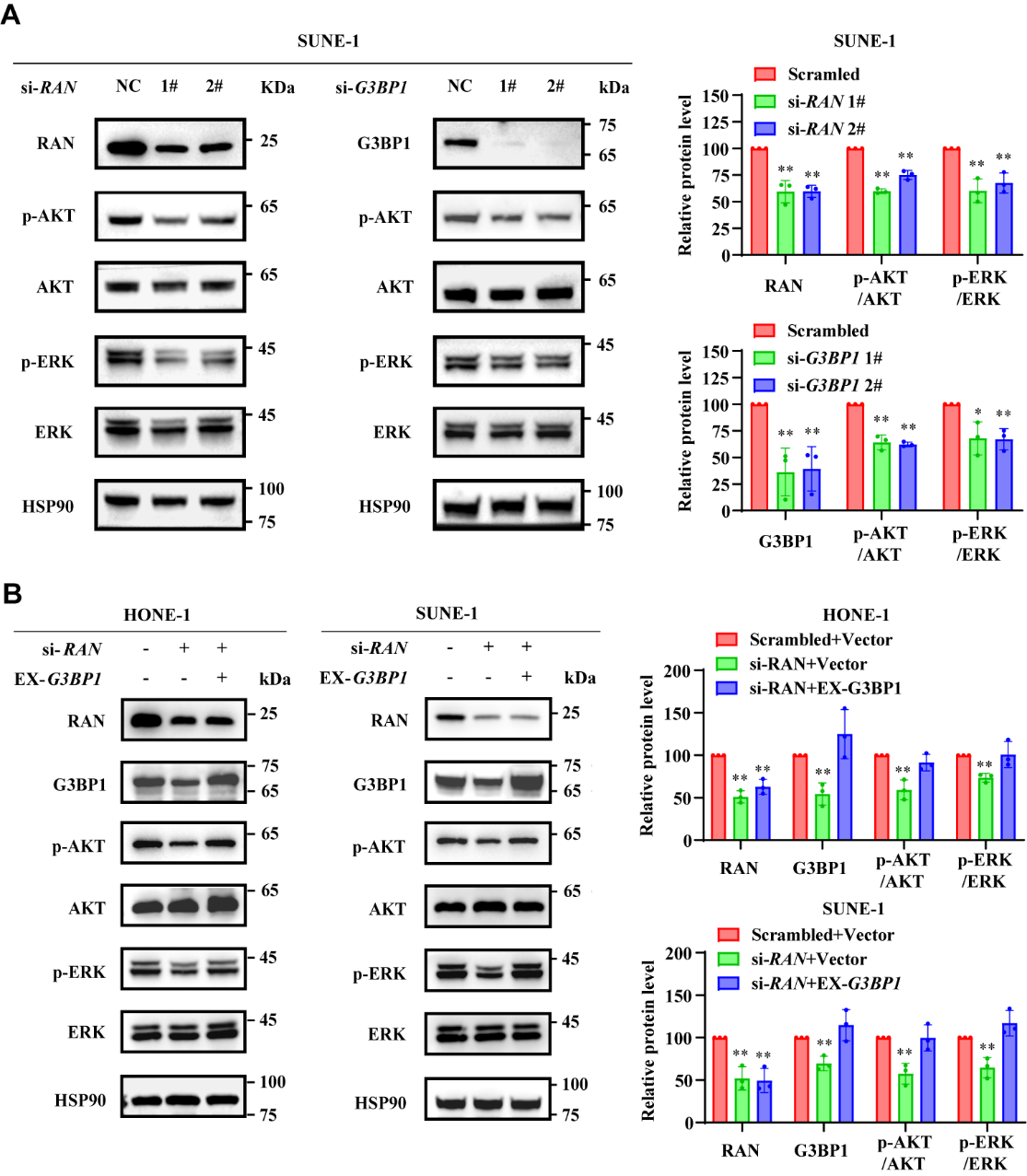
**

**Supplementary Figure S10.** **RAN facilitates AKT/ERK phosphorylation via G3BP1.** A, AKT, p-AKT, ERK, and p-ERK protein levels with RAN or G3BP1 knockdown were detected by Western blotting in SUNE-1 cells. The blots are the representation of three independent experiments. Data are presented as the mean ± SD (n=3). B, p-AKT, and p-ERK protein levels were detected by Western blotting in HONE-1 and SUNE-1 cells after co-transfected with scrambled control or si-RAN, together with empty vector or G3BP1 overexpression vector. The blots are the representation of three independent experiments. Data are presented as the mean ± SD (n=3). **P* < 0.05, ***P* < 0.01. The significant differences were assessed using one-way ANOVA (A, B).
