# Supplementary material for: The noncanonical RNA-binding protein RAN stabilizes the mRNA of intranuclear stress granule assembly factor G3BP1 in nasopharyngeal carcinoma
Source: J Biol Chem. 2024 Nov 5;300(12):107964. doi: 10.1016/j.jbc.2024.107964 (PMC11635782; doi:10.1016/j.jbc.2024.107964)
Supplement: Supplemental Table S1–S6 [file mmc2.docx]

**Supplementary Tables: 6**

**Supplementary Table S1.** siRNA sequence used in this study

| **Name** | **Sequence (5’ to 3’)** |
| --- | --- |
| si-ALYREF-1#-F | GGCGGCUGUGCACUAUGAUTT |
| si-ALYREF-1#-R | AUCAUAGUGCACAGCCGCCTT |
| si-ALYREF-2#-F | GCUUGUCACGUCACAGAUUTT |
| si-ALYREF-2#-R | AAUCUGUGACGUGACAAGCTT |
| si-C1QBP-1#-F | GGCCUUAUAUGACCACCUATT |
| si-C1QBP-1#-R | UAGGUGGUCAUAUAAGGCCTT |
| si-C1QBP-2#-F | GAGAGUGACAUCUUCUCUATT |
| si-C1QBP-2#-R | UAGAGAAGAUGUCACUCUCTT |
| si-EZH2-1#-F | GAGGGAAAGUGUAUGAUAATT |
| si-EZH2-1#-R | UUAUCAUACACUUUCCCUCTT |
| si-EZH2-2#-F | GACUCUGAAUGCAGUUGCUTT |
| si-EZH2-2#-R | AGCAACUGCAUUCAGAGUCTT |
| si-G3BP1-1#-F | GGGCUUCUCUCUAACAACATT |
| si-G3BP1-1#-R | UGUUGUUAGAGAGAAGCCCTT |
| si-G3BP1-2#-F | GCGAGAACAACGAAUAAAUTT |
| si-G3BP1-2#-R | AUUUAUUCGUUGUUCUCGCTT |
| si-HRSP12-1#-F | GGGUGAUCAGCACCGCGAATT |
| si-HRSP12-1#-R | UUCGCGGUGCUGAUCACCCTT |
| si-HRSP12-2#-F | CCCUACAGUCAAGCUGUAUTT |
| si-HRSP12-2#-R | AUACAGCUUGACUGUAGGGTT |
| si-MAGOHB-1#-F | GGACGGAAAGCUUAGAUAUTT |
| si-MAGOHB-1#-R | AUAUCUAAGCUUUCCGUCCTT |
| si-MAGOHB-2#-F | GGAAGAACUGAAGAGAAUUTT |
| si-MAGOHB-2#-R | AAUUCUCUUCAGUUCUUCCTT |
| si-NCBP2-1#-F | CCAGGUUCGGGAUGAGUAUTT |
| si-NCBP2-1#-R | AUACUCAUCCCGAACCUGGTT |
| si-NCBP2-2#-F | CAGAUUGGAUCUGUAAUGUTT |
| si-NCBP2-2#-R | ACAUUACAGAUCCAAUCUGTT |
| si-RAN-1#-F | GGAGACCCUAACUUGGAAUTT |
| si-RAN-1#-R | AUUCCAAGUUAGGGUCUCCTT |
| si-RAN-2#-F | CGUCAUUUGACUGGUGAAUTT |
| si-RAN-2#-R | AUUCACCAGUCAAAUGACGTT |
| si-RAN-3' UTR-1#-F | GCGUGUGUGCCACCUCAUUTT |
| si-RAN-3' UTR-1#-R | AAUGAGGUGGCACACACGCTT |
| si-RAN-3' UTR-2#-F | GCUUAGUGUGAAGUUGAUATT |
| si-RAN-3' UTR-2#-R | UAUCAACUUCACACUAAGCTT |
| si-RDM1-1#-F | GCGAAUUACUACUUUGGUUTT |
| si-RDM1-1#-R | AACCAAAGUAGUAAUUCGCTT |
| si-RDM1-2#-F | UCAGAAGGCUUUGUCAGAUTT |
| si-RDM1-2#-R | AUCUGACAAAGCCUUCUGATT |
| si-RNASEH2A-1#-F | GGUCUACGCCAUCUGUUAUTT |
| si-RNASEH2A-1#-R | AUAACAGAUGGCGUAGACCTT |
| si-RNASEH2A-2#-F | GGGUCAAAUACAACCUGAATT |
| si-RNASEH2A-2#-R | UUCAGGUUGUAUUUGACCCTT |
| si-SF1-1#-F | GCAACACGUGUGAGUGAUATT |
| si-SF1-1#-R | UAUCACUCACACGUGUUGCTT |
| si-SF1-2#-F | GGAACACCCUGAAGAACAUTT |
| si-SF1-2#-R | AUGUUCUUCAGGGUGUUCCTT |
| si-TDP43-1#-F | CCGGCUGGUAGAAGGAAUUTT |
| si-TDP43-1#-R | AAUUCCUUCUACCAGCCGGTT |
| si-TDP43-2#-F | GGAGUAUAUUCAGCAGUAUTT |
| si-TDP43-2#-R | AUACUGCUGAAUAUACUCCTT |

**Supplementary Table S2.** qRT-PCR primers used in this study

| **Gene** | **Sequence (5’ to 3’)** |
| --- | --- |
| ALYREF-F | GGAGTCTCAGACGCCGATATTC |
| ALYREF-R | GCATCTGCCTTCCGCTCAAAGT |
| ANXA2-F | TCGGACACATCTGGTGACTTCC |
| ANXA2-R | CCTCTTCACTCCAGCGTCATAG |
| BZW1-F | TGCAGTAGCTGCAAGTCTTCGG |
| BZW1-R | CTCCGATGGTTTGCTGATTCCG |
| C1QBP-F | CTTTCAGTCCACTGGCGAGTCT |
| C1QBP-R | CGGCAAGGAAATCCATTAGGTGG |
| CDCP1-F | CAGGTGAAGCAGAACATCTCGG |
| CDCP1-R | GTCACCGTGAAAACGCCTTCCT |
| EZH2-F | GACCTCTGTCTTACTTGTGGAGC |
| EZH2-R | CGTCAGATGGTGCCAGCAATAG |
| G3BP1-F | AGCCTGTTCAGAAAGTCCTTAGC |
| G3BP1-R | CGAAGGCGATTATCTCGTCGGT |
| GAPDH-F | TGATGACATCAAGAAGGTGG |
| GAPDH-R | TTGTCATACCAGGAAATGAGC |
| HRSP12-F | CTGAAAGCTGCAGGCTGTGACT |
| HRSP12-R | GCAACTTGGTAAGCAGCTCTAGC |
| HPCAL1-F | CCTTCAGCATGTACGACCTGGA |
| HPCAL1-R | GATCTTGTCTGTGCGCTTCTCC |
| ITGA6-F | CGAAACCAAGGTTCTGAGCCCA |
| ITGA6-R | CTTGGATCTCCACTGAGGCAGT |
| MAGOHB-F | TCTGGAGTTCGAATTTCGGCCG |
| MAGOHB-R | CTCTTCAGTTCTTCCATTACACTC |
| MALL-F | CCGTCCTACAAGTACATGCCAC |
| MALL-R | TGTAGAGCAGCGTGGCGATGAA |
| MCL1-F | CCAAGAAAGCTGCATCGAACCAT |
| MCL1-R | CAGCACATTCCTGATGCCACCT |
| NCBP2-F | CCGAATCATTCGCACAGACTGG |
| NCBP2-R | CTGTGCCAGTTTTCCATAGCCTC |
| PKM-F | ATGGCTGACACATTCCTGGAGC |
| PKM-R | CCTTCAACGTCTCCACTGATCG |
| PABPC1-F | CAGAGAATGGCAAGTGTACGAGC |
| PABPC1-R | GCTAGGAGGATAGTATGCAGCAC |
| PGAM1-F | GCTCTGCCCTTCTGGAATGAAG |
| PGAM1-R | ATACCAGTCGGCAGGTTCAGCT |
| RAN-F | CCACCAGAAGTTGTCATGGACC |
| RAN-R | CTCCAGCTTCATTCTCACAGGTC |
| RDM1-F | GGCAGTTCAACATCAAGCCCTTG |
| RDM1-R | CTTCGGAAGTGGCACCATGCTA |
| RNASEH2A-F | GCCGTGAAGAAATGGCAGTTCG |
| RNASEH2A-R | GTGCTCCTTCAACCACGCTTTTG |
| RPL8-F | CCGTATCGGTTTAAGAAGCGGAC |
| RPL8-R | CGATTGTACCCTCAGGCATGGT |
| RPL4-F | ACGATACGCCATCTGTTCTGCC |
| RPL4-R | GGAGCAAAACAGCTTCCTTGGTC |
| SF1-F | CCGCAGCATTACCAACACCACA |
| SF1-R | CATCCGTGCTTTATCCTGAGCTG |
| SFN-F | TGCTGGACAGCCACCTCATCAA |
| SFN-R | GGCTGAGTCAATGATGCGCTTC |
| TDP43-F | GATGGACGATGGTGTGACTGCA |
| TDP43-R | AAGAACTCCCGCAGCTCATCCT |
| TM9SF3-F | TGCTTCAAGAGCCATTCCTTTTGG |
| TM9SF3-R | CACGACAAGGAAAGTTGGGCTG |
| U3-F | TAGAGCACCGAAAACCACGA |
| U3-R | CCTCTCACTCCCCAATACGG |
| U6-F | CTCGCTTCGGCAGCACATATACT |
| U6-R | ACGCTTCACGAATTTGCGTGTC |
| YWHAZ-F | ACCGTTACTTGGCTGAGGTTGC |
| YWHAZ-R | CCCAGTCTGATAGGATGTGTTGG |
| 18S rRNA-F | CAGCCACCCGAGATTGAGCA |
| 18S rRNA-R | TAGTAGCGACGGGCGGTGTG |

**Supplementary Table S3.** Primers used for shRNA plasmid construction

| **Name** | **Sequence (5’ to 3’)** |
| --- | --- |
| sh-RAN-1#-F | CCGGACGTCATTTGACTGGTGAATTCTCGAGAATTCACCAGTCAAATGACGTTTTTTG |
| sh-RAN-1#-R | AATTCAAAAAACGTCATTTGACTGGTGAATTCTCGAGAATTCACCAGTCAAATGACGT |
| sh-RAN-2#-F | CCGGGACCCTAACTTGGAATTTGTTCTCGAGAACAAATTCCAAGTTAGGGTCTTTTTG |
| sh-RAN-2#-R | AATTCAAAAAGACCCTAACTTGGAATTTGTTCTCGAGAACAAATTCCAAGTTAGGGTC |
| sh-RAN-3#-F | CCGGGCACAGTATGAGCACGACTTACTCGAGTAAGTCGTGCTCATACTGTGCTTTTTG |
| sh-RAN-3#-R | AATTCAAAAAGCACAGTATGAGCACGACTTACTCGAGTAAGTCGTGCTCATACTGTGC |

**Supplementary Table S4.** Primers sequence used for RIP-PCR assay

| **Name** | **Sequence (5’ to 3’)** |
| --- | --- |
| G3BP1-RIP-F1 | AGCCTGTTCAGAAAGTCCTTAGC |
| G3BP1-RIP-R1 | CGAAGGCGATTATCTCGTCGGT |
| G3BP1-RIP-F2 | CACCTGCAGACATAGCTCA |
| G3BP1-RIP-R2 | TTTATTCGTTGTTCTCGCACT |
| G3BP1-RIP-F3 | ACTTTAATATTGGACTTTGCTC |
| G3BP1-RIP-R3 | GGCCATTTCCAAGTAACAC |

**Supplementary Table S5.** Clinical characteristics of the cohort of nasopharyngeal carcinoma patients (n=211).

| **Characteristics** | **No. of patients** | **No. (%)** |
| --- | --- | --- |
| **Age** |  |  |
| <45 | 104 | 49.3 |
| ≥45 | 107 | 50.7 |
| **Sex** |  |  |
| Male | 163 | 77.3 |
| Female | 48 | 22.7 |
| **T category** |  |  |
| T1-T2 | 72 | 34.1 |
| T3-T4 | 139 | 65.9 |
| **N category** |  |  |
| N0-N1 | 129 | 61.1 |
| N2-N3 | 82 | 38.9 |
| **TNM Stage** |  |  |
| Ⅰ-III | 131 | 62.1 |
| IV | 80 | 37.9 |
| **Pathological type** |  |  |
| WTO Ⅰ | 0 | 0 |
| WTO II | 0 | 0 |
| WTO III | 211 | 100 |
| **Treatment** |  |  |
| Radiotherapy alone | 15 | 7.1 |
| Concurrent chemoradiotherapy | 77 | 36.5 |
| Induction chemotherapy +  Radiotherapy alone | 6 | 2.8 |
| Induction chemotherapy +  Concurrent chemoradiotherapy | 99 | 46.9 |
| Radiotherapy alone +  Adjuvant chemotherapy | 0 | 0 |
| Concurrent chemoradiotherapy +  Adjuvant chemotherapy | 5 | 2.4 |
| Induction chemotherapy +  Radiotherapy alone +  Adjuvant chemotherapy | 2 | 0.9 |
| Induction chemotherapy +  Concurrent chemoradiotherapy +  Adjuvant chemotherapy | 7 | 3.3 |

**Supplementary Table S6.** Clinical characteristics of nasopharyngeal carcinoma patients according to the high and low expression of RAN

| **Characteristics** | **No. of patients** | **Expression of RAN** | | ***P* value** |
| --- | --- | --- | --- | --- |
|  |  | **Low, *n* (%)** | **High, *n* (%)** |  |
| **Age** |  |  |  | 0.169 |
| <45 | 104 | 59 (54.1) | 45 (44.1) |  |
| ≥45 | 107 | 50 (45.9) | 57 (55.9) |  |
| **Sex** |  |  |  | 0.327 |
| Male | 163 | 81 (74.3) | 82 (80.4) |  |
| Female | 48 | 28 (25.7) | 20 (19.6) |  |
| **T category** |  |  |  | **0.006** |
| T1-T2 | 72 | 47 (43.1) | 25 (24.5) |  |
| T3-T4 | 139 | 62 (56.9) | 77 (75.5) |  |
| **N category** |  |  |  | 0.259 |
| N0-N1 | 129 | 71 (65.1) | 58 (56.9) |  |
| N2-N3 | 82 | 38 (34.9) | 44 (43.1) |  |
| **TNM Stage** |  |  |  | **0.047** |
| Ⅰ-III | 131 | 75 (68.8) | 56 (54.9) |  |
| IV | 80 | 34 (31.2) | 46 (45.1) |  |
| **Pathological type** |  |  |  |  |
| WTO Ⅰ | 0 | 0 | 0 |  |
| WTO II | 0 | 0 | 0 |  |
| WTO III | 211 | 109 (100) | 102 (100) |  |
| **Treatment** |  |  |  | 0.634 |
| Radiotherapy alone | 15 | 8 (7.3) | 7 (6.9) |  |
| Concurrent chemoradiotherapy | 77 | 40 (36.7) | 37 (36.3) |  |
| Induction chemotherapy +  Radiotherapy alone | 6 | 3 (2.8) | 3 (2.9) |  |
| Induction chemotherapy +  Concurrent chemoradiotherapy | 99 | 53 (48.6) | 46 (45.1) |  |
| Radiotherapy alone +  Adjuvant chemotherapy | 0 | 0 | 0 |  |
| Concurrent chemoradiotherapy +  Adjuvant chemotherapy | 5 | 3 (2.8) | 2 (2.0) |  |
| Induction chemotherapy +  Radiotherapy alone +  Adjuvant chemotherapy | 2 | 1 (0.9) | 1 (1.0) |  |
| Induction chemotherapy +  Concurrent chemoradiotherapy +  Adjuvant chemotherapy | 7 | 1 (0.9) | 6 (5.9) |  |

All patients were restaged according to the 8th edition of the AJCC Cancer Staging Manual. Bold values indicate *P* < 0.05, P value is determined by χ^2^ and Fisher’s exact tests.
